# Structure determination and dual targeting of a plant TACO1 identifies its ancient role as an organelle translation regulator

**DOI:** 10.64898/2026.05.28.728419

**Published:** 2026-06-01

**Authors:** Parth K. Raval, Nuša Kos Thaler, Cristopher Mitchell, Maria Lozano-Quiles, Tommi Kajander, Daniel W. Djamriani, Jens Reiners, Sander HJ Smits, Sarah J. Butcher, Brendan J. Battersby, Sven B. Gould

**Affiliations:** 1 Institute for Molecular Evolution, Heinrich-Heine-Universität Düsseldorf, 40225 Düsseldorf, Germany; 2 Helsinki Institute of Life Science-Institute of Biotechnology, University of Helsinki, Helsinki, Finland; 3 Department of Molecular and Integrative Biosciences, Faculty of Biological and Environmental Sciences, University of Helsinki, Helsinki, Finland; 4 Center for Structural Studies, Heinrich-Heine-Universität Düsseldorf, 40225 Düsseldorf, Germany; 5 Institute for Population Genetics, Heinrich-Heine-Universität Düsseldorf, 40225 Düsseldorf, Germany

**Keywords:** YebC, TACO1, ribosome stalling, polyproline motifs, organelle translation

## Abstract

Ribosome stalling caused by polyproline (PPs) motifs is common. Their translation is enhanced by accessory proteins such as YebC in bacteria, whose homolog, TRANSLATIONAL ACTIVATOR OF CYTOCHROME C OXIDASE 1 (TACO1), aids the translation of mitochondria-encoded proteins. The prevalence of PP motifs across plastid-encoded genes and their impact on the translation of photosynthesis-relevant proteins remains unexplored. Equally, a translation-enhancer of PP motifs equivalent to TACO1 for plastid ribosomes has not been reported. Here, we show that plastid genomes encode 24 proteins with a minimum of one PP motif on average, half of which are conserved in their cyanobacterial homologs, and that the vast majority of eukaryotes, including plants, encode a single TACO1 that we demonstrate to be dually targeted to mitochondria and plastids of *Marchantia polymorpha.* We resolved the MpTACO1 structure at 2.34 Å by X-ray crystallography and the flexibility by small-angle X-ray scattering. Through modelling, we demonstrate that MpTACO1 can fit into the peptidyl transfer centre of plant chlororibosomes in a similar manner as human TACO1 in the mitoribosome. The identification and structure determination of the first plastid-targeted YebC/TACO1 allows us to sketch a unified model for the function and evolution of this ancient family of ribosomal accessory proteins, underscoring their indispensable role in the translation of bioenergetic membrane proteins reaching back almost 4 billion years.

## Introduction

Plant cells are the product of serial endosymbiosis. The timing of the events remains uncertain, but occurred in the Palaeoproterozoic era^1^ and first gave rise to eukaryotes through the origin of mitochondria, and subsequently to phototrophic eukaryotes through the origin of the plastid^2–6^. The transition from a bacterial endosymbiont to a eukaryotic organelle entails the transfer of bacterial genes to the nucleus through endosymbiotic gene transfer (EGT)^7–10^. Thousands of organellar proteins are hence translated in the cytosol and must be imported by the organelles of endosymbiotic origin^11–14^. Despite EGT and the emergence of dedicated protein import systems, both mitochondria and plastids still encode a small, albeit key, subset of genes that are translated into proteins in their matrix and stroma, respectively.

For both organelle genomes, one can sort most of their genes into those encoding either the remaining subunits of the ribosomes or proteins of the oxidative phosphorylation complexes of mitochondrial respiration and plastid photosynthesis^15–17^. Some ribosomal proteins remain organelle-encoded due to functional constraints associated with ribosomal complex assembly^16^, while bioenergetic membrane protein-encoding genes are likely retained due to issues associated with their hydrophobicity^18–21^ and *in organello* redox regulation^22–24^. Organelle-encoded complexes require nuclear-encoded subunits to function, some of which are dual targeted to both organelles after the translation from a single gene, e.g. rpL10 or rpS16^25,26^. The maturation of bioenergetic membrane complexes is hence a delicate process that requires a spatiotemporal orchestrated assembly of factors encoded by two genomes. The principal components and the core mechanism of protein translation and membrane insertion were inherited by eukaryotic organelles from their bacterial ancestors and remain well conserved^27,28^.

Ribosomes, whether cytosolic, mitochondrial or plastid, can stall during translation elongation due to mRNA structures such as hairpins or challenging amino acid sequences, most notably proline residues^25,29–35^. Proline’s unique cyclic structure reduces conformational flexibility during the formation of peptide bonds, which can form trans- and cis-configurations at the peptidyl transferase center (PTC) of the ribosome, resulting in significantly slower translation rates^35,36^. Several accessory factors evolved early in evolution to aid the peptide bond formation with prolines and prevent stalling, specifically for the translation of two or more consecutive prolines (hereafter referred to as polyproline or PP motifs). These include the bacterial Elongation Factor P (EF-P), the archaeal Initiation Factor 5A (aIF-5A), members of the ABC-F ATPases, and YebC^37,38^. In mitochondria, a homolog of YebC called TRANSLATIONAL ACTIVATOR OF CYTOCHROME C OXIDASE 1 (TACO1) is required for the mitochondrial synthesis of COX1 and, potentially also COX3^39–42^. Human COX1 encodes no less than five PP motifs of which at least three have been found to stall ribosomes^39^. Recent *in organello* cryogenic electron microscopy (cryoEM) found TACO1 interacting with the mitochondrial 16S rRNA at the mRNA entry site, bridging the small and large mitoribosomal subunits^43^. YebC enhances the translation of polyproline stretches in bacteria^37^ and was recently shown to transiently interact with the bacterial 23S rRNA of the 70S ribosome^38^. A model is emerging whereby factors such as YebC and its eukaryotic homolog TACO1 stabilize the A-site prolyl-tRNA during peptide bond formation on bacterial and mitochondrial ribosomes.

**Fig. 1:**
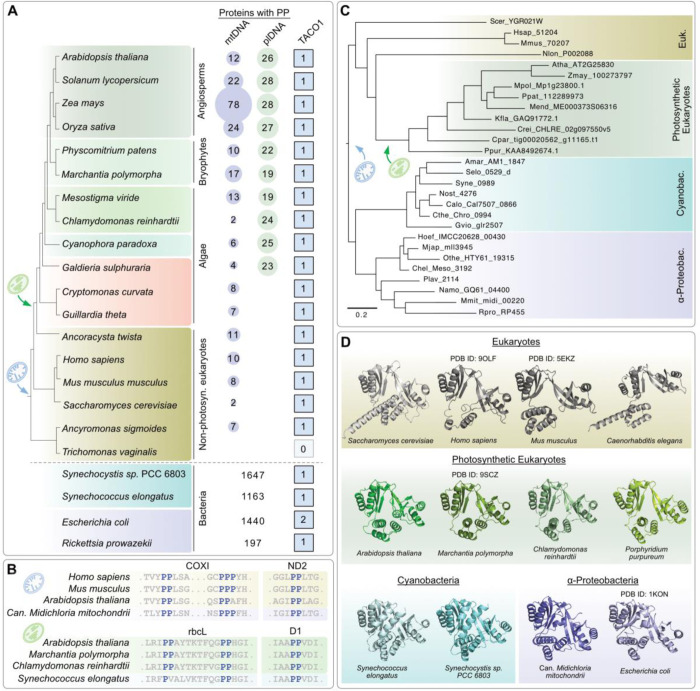
Conservation of YebC/TACO1 homologs and PP motifs across bacteria and eukaryotes. **(A)** Number of polyproline (PP) motifs containing proteins in bacterial, mitochondrial and plastid genomes along with the number of genome-encoded TACO homologs across diverse eukaryotes and prokaryotes. **(B)** Examples of PP motifs that are present in alpha-proteobacteria and cyanobacteria, and which were inherited by eukaryotes through endosymbiosis and remain organelle-encoded. **(C)** Phylogenetic tree of YebC/TACO1 genes, rooted between prokaryotes and eukaryotes. **(D)** Experimentally resolved (human PDB ID:9OLF, mouse PDB ID:5EKZ, *E. coli* PDB ID:1KON, and in this study *M. polymorpha* PDB ID:9SCZ) and predicted AlphaFold3^47^ structures of YebC/TACO1 proteins from selected diverse eukaryotes and prokaryotes.

Plastids are of cyanobacterial origin and like most bacteria, cyanobacteria encode a YebC homolog (Fig. 1, Fig. S1). There are, however, neither reports of the cyanobacterial YebC’s fate in algae and plants, nor the prevalence and potential impact of prolines and PP motifs on protein translation in plastids. This presents us with an unexplored phenomenon of plastid biology. Here, we screened 15,208 algae and plant plastid genomes and found that, on average, every genome encodes a quarter of the genes with at least one PP motif. Of the 169 nuclear genomes of photosynthetic and non-photosynthetic eukaryotes we screened, 122 encode only a single YebC/TACO1 homolog, which we demonstrate is dual targeted to mitochondria and plastids of the liverwort *Marchantia polymorpha*. We solved the crystal structure of *M. polymorpha* TACO1 (MpTACO1), revealing a highly conserved structure, and further using SAXS revealed three conformations in solution. Our data suggest that the plant YebC/TACO1 homolog is a conserved protein critical for the translation regulation of organelle-encoded proteins not only in mitochondria, but also in the plastids of algae and plants.

## Results

### Organelle-encoded PP motifs and YebC/TACO1 proteins share an ancient history

While PP motifs are sporadically discussed for individual proteins or a species group, their global frequency and the level of conservation across distant taxa has not been explored, especially not for plastid genomes. We screened 18,059 mitochondrial and 15,208 plastid genomes of diverse eukaryotes. Mitochondrial genomes encode six proteins with at least one PP motif on average (42.6% of the organelle-encoded genes), while plastid genomes encode 24 (29.4%) (Fig. 1A, Fig. S1A). The positions of many PP motifs are evolutionarily conserved in bacteria, animals and plants (Fig. 1B, Fig. S2-3) and includes motifs of COXI and the photosystem II protein D1 (psbA) that are found in the vicinity of transmembrane domains. As prolines are encoded by the CCN codon, this speaks for a >1.5 billion years long retention of these GC-rich stretches by plastid genomes that evolve under evolutionary pressures that favour AT-rich genomes ^44,45^. Despite that pressure, 56 diverse alpha-proteobacterial and 63 cyanobacterial genomes encode about a thousand PP-containing proteins each (Fig. 1A, Fig. S1A).

The search for YebC/TACO1 homologs among the genomes of 113 bacteria and 169 eukaryotes finds that 107 (94.6%) of the prokaryotes and 122 (72.1%) of the eukaryotes encode only a single copy (Fig. 1A, Fig. S1B). Eukaryotic species with more than one copy include a small subset of plants with extreme polyploidy and early-branching eukaryotes, for which their draft genomes returned only partial YebC/TACO1-like sequences (Fig. S1A). In any case, a majority of land plant species with extreme ploidy and those that are otherwise characterised by an expansion of organelle-targeted proteins^46^, encode only a single YebC/TACO1 protein. Eukaryotes lacking YebC/TACO1 homologs include parasites such as *Giardia intestinalis* and *Trichomonas vaginalis,* whose mitochondria-derived organelles lack genomes and hence do not translate proteins, underscoring YebC/TACO1’s role as an accessory protein of mitoribosomes which trace their ancestry to bacterial ribosomes.

**Fig. 2:**
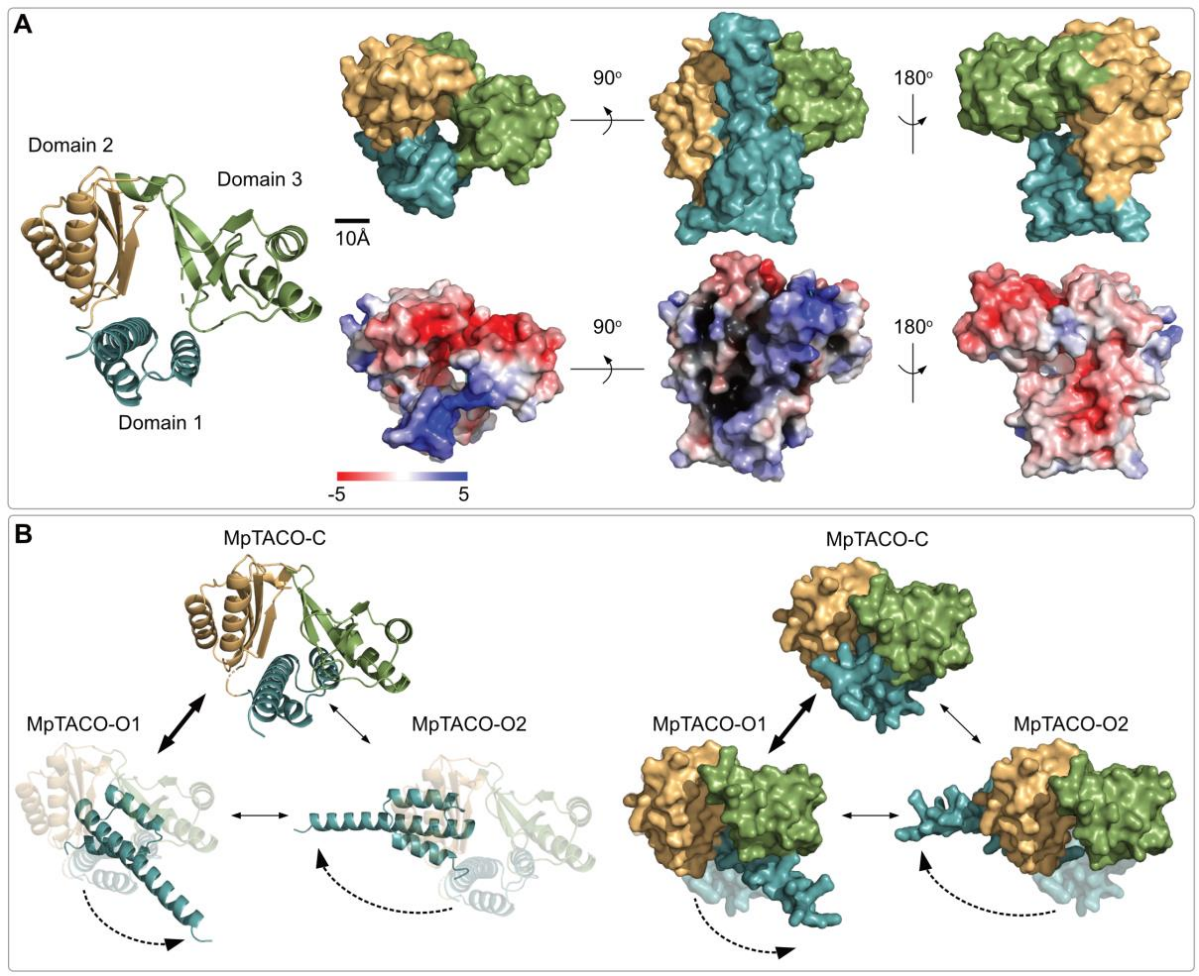
MpTACO1 contains a conserved hinge and forms three distinct conformations in solution. **(A)** Crystal structure of MpTACO1 represented as a ribbon-based cartoon, along with surface views colour-coded by domains (top) and by local electrostatic potentials (bottom). **(B)** Three conformations present within the ensemble at 40%, 50% and 10%, labelled MpTACO-Closed (MpTACO-C), MpTACO-Open1 (MpTACO-O1) and MpTACO-Open2 (MpTACO-O2). MpTACO-C resembles the most compact structure solved from X-ray crystallography and is shown as a semi-transparent structure underneath O1 and O2 conformations to indicate the relative motion of domain 1. Arrow thickness indicates the dominance with which the conformational change was measured.

A phylogenetic analysis places all prokaryotic YebC proteins from both alphaproteobacteria and cyanobacteria – representing the donor groups to the endosymbiotic origin of mitochondria and plastids, respectively – as a monophyletic sister clade to eukaryotic TACO1 sequences, where algae and plant TACO1 homologs form a monophyletic group (Fig.1C). Due to the high level of sequence conservation, a definite claim on whether the alphaproteobacterial (mitochondrial) or cyanobacterial (plastid) YebC has been retained in plants is hence not possible. The sequence conservation is echoed by the predicted and experimentally validated structures of YebC/TACO1 proteins from across the tree of life (Fig. 1C-D, Fig.2, S4A). Structure-based dendrograms and clustering also place prokaryotic homologs together as a monophyletic sister clade to eukaryotes (Fig. S4B-D). Taken together, our analyses underscore an ancient and abundant occurrence of PP motifs across prokaryotic genomes, some of which remain encoded by the two organelles of endosymbiotic origin, and that a single TACO1 protein associated with their translation, at least in mitochondria, is present in the vast majority of eukaryotes, including algae and plants.

### X-ray crystallography and SAXS uncovers three distinct conformations of MpTACO1

Thus far, the worldwide protein data bank houses five YebC/TACO1 family atomic models for bacteria and mammals, with no entries for plants. To fill this gap, we recombinantly expressed the Marchantia homolog MpTACO1 (Mp1g23800; coding for amino acids 127–374) in *E. coli.* Amino acids 1–126 were omitted because a protein alignment of eukaryotic TACO1 and bacterial YebC sequences suggested the N-terminal targeting peptide (TP) to be at least 100 amino acids long (Fig. S5A). TPs tend to be unstructured, can interfere with crystallisation and offer no information on the function of a mature protein. MpTACO1 lacking the predicted targeting sequence was purified as a monomer, crystallized, and we determined its structure with X-ray diffraction using molecular replacement (Fig. S5B-F). We obtained a resolution of 2.34 Å with 97.61% completeness, which allowed for the atomic modelling of residues 127–293 and 301–373, comprising the fully encoded construct except for 7 residues in a flexible loop region of domain 3, with no Ramachandran or rotamer outliers, (Fig. S5, Table S1), which together confirms the high quality of the model. MpTACO1 comprises three domains (Fig. 2A): domain 1 (residues 127–200,) consists of a bundle of three α-helices, domain 2 (residues 201–253, 339–373) features three antiparallel β-strands facing two α-helices, and domain 3 (residues 254–338) contains four antiparallel β-strands that face two α-helices (Fig. 2A). The surface of domain 1 is primarily positively-charged, domain 2 has mixed charges, and domain 3 is primarily negatively-charged (Fig. 2A).

**Fig. 3:**
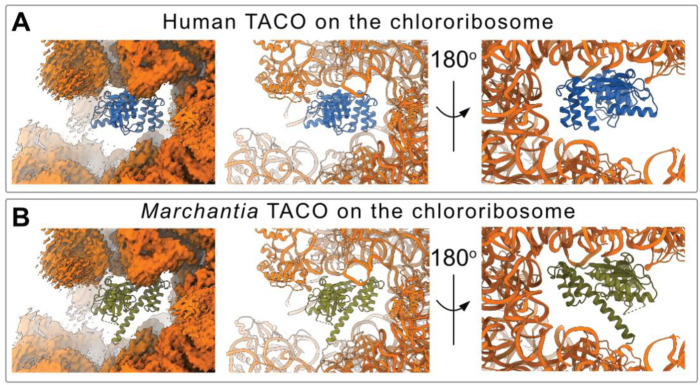
The entry site of the chlororibosome fits both the human and plant TACO1 homologs. Chloroplast ribosome from spinach (PDB: 5MMM) contains a pocket at the mRNA entry site, where both **(A)** the human (PDB: 9OLF) and **(B)** the *M. polymorpha* TACO1 protein (PDB: 9SCZ) can fit. The isosurface representation of the chlororibosome is shown at 3 **σ** above the mean (the leftmost panels) and as cartoons. This region is analogous to the experimentally characterized TACO1 binding pocket of human mitoribosomes^43^.

The structure of TACO1 homologs has been reported to exhibit both an open and closed cleft between domains 2 and 3, previously thought to be an RNA-binding cleft, suggesting that conformational changes are possible^40,48,49^. To analyse the behaviour of MpTACO1 in solution, we performed small-angle X-ray scattering (SAXS) experiments. The SAXS results also showed that MpTACO1 is a monomer in solution (see Table S2). Initially, the crystal structure of MpTACO1 agreed with the experimental SAXS data (χ^2^ value 1.39), however, the residual plot indicated that the domains needed to be rearranged to fit the experimental data (Fig. 2B). Based on the Kratky plot, MpTACO1 is a compact molecule with a certain degree of flexibility, which is consistent with the notion of open and closed conformations suggested from previous studies (Fig. S6). To analyse this movement in solution, we used an Ensemble Optimisation Method (EOM). In this analysis, domain 1 is free to move in solution and the resulting $R_{g}$ distribution primarily exhibits two volume fractions with $R_{g}$ values of 2.01 nm and 2.11 nm, respectively (Fig. 2B, Fig. S6). The protein maintains an overall compact conformation, however, representing closed (MpTACO-C, volume fraction 40%) and open conformations (MpTACO-O1, volume fraction 40% and MpTACO-O2, volume fraction 10%) within the ensemble (Fig. 2B, Fig. S6), with the closed conformation being similar to that of the crystal structure and to that of the human TACO1 modelled in the bound state to human mitoribosomes^43^. We thus modelled MpTACO1 into a spinach plastid ribosome structure^50^ and showed that it can indeed fit into a similar location (Fig. 3), despite the extended N-terminal helix in domain 1. On closer inspection of the published density for human TACO1 bound to the human mitoribosome (EMD-70592), it was evident that the occupancy of domain 1 was very weak, close to the noise level, compared to the other two domains that can be easily distinguished at 2 standard deviations (σ) above the mean^43^. This supports our observations of domain 1 being flexible relative to the other two domains.

### MpTACO1 is dual targeted to mitochondria and plastids

**Fig. 4:**
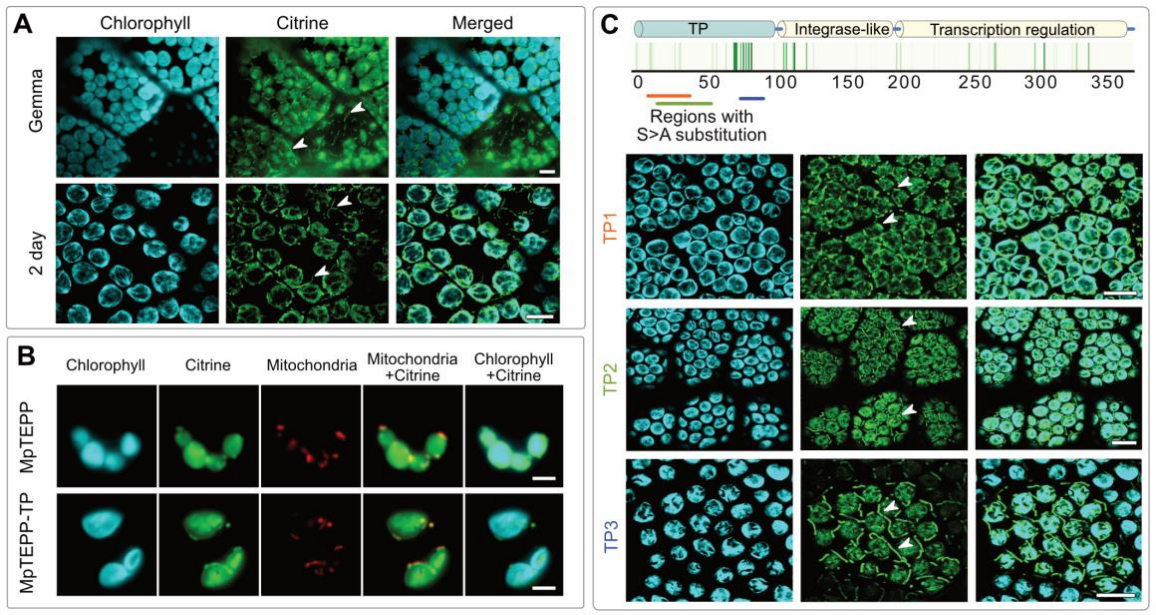
Protein dual targeting of TACO1 in the liverwort *Marchantia*. **(A)** Intracellular localisation of MpTACO1 in gemmae and two-day old thalli of *M. polymorpha.* **(B)** Images of protoplasts generated from thalli of one week old isogenic plant lines expressing both full MpTACO1 and only its targeting peptide (TP) fused to citrine. **(C)** Likelihood of serine phosphorylation in the TP of MpTACO1 depicted by shades of green, where each bar represents an amino acid residue. Key serine residues were mutated to alanine in three constructs: TP1 shown in orange (substituted residues: 4, 5, 14–17, 33, 36, 37), TP2 in green (substituted residues: 4, 5, 14–17, 33, 36, 37, 52, 54, 55), and TP3 in blue (substituted residues: 77–79, 82, 84, 86). Average phosphorylation probabilities of the mutated serine motifs were 0.17 (TP1), 0.32 (TP2) and 0.71 (TP3). Plastids were imaged through their autofluorescence and protoplast mitochondria through MitoTracker Red. Reticulate mitochondria are indicated by white arrow heads. Scalebars 10μm.

We fused the full-length sequence of MpTACO1 to citrine and determined its localization *in vivo* in *M. polymorpha*. The construct was targeted to mitochondria and plastids and the citrine signal appeared to be equally distributed between both compartments in the gemmae and adult thalli stages of the liverwort (Fig. 4A, Fig. S7A. Fig. S8). As with the full-length sequence, the first 105 amino acids of MpTACO1 were sufficient to sort the reporter to both mitochondria and plastids in protoplasts, which also allow for a better visualization of stained mitochondria (Fig. 4B, Fig. S7B, Fig. S9). Finally, we isolated plastids from two independent transfectants and validated the presence of the full-length construct through immunoblotting (Fig. S10).

Plastid targeting peptides (pTPs) are usually enriched in amino acids that can be phosphorylated and the more N-terminal regions of mitochondrial TPs in positive charges ^11,51^. Dual targeted proteins require a balance of both these features and indeed the TP of MpTACO1 encodes 22 serine residues with a mean phosphorylation probability of 0.45 and the first 25 amino acids have an overall charge of +4 (Fig. 4C). The abundance of serine residues suggests a degree of redundancy that may maintain and/or regulate dual targeting. To probe this, we substituted selected serine with alanine residues and generated three constructs, where 6–12 key serine residues were altered in total. Dual targeting to mitochondria and plastids was maintained by all three constructs, even upon the substitution of twelve serine residues as in construct TP2 (Fig. 4C, S7C). It suggests that MpTACO1 evolved a TP that can maintain dual targeting robustly, suggesting a crucial role in both organelles. Next to TACO1, we show that another three proteins associated with transcription and translation are dual targeted in Marchantia (Fig. S11), which corroborated the frequent dual localisation of information-processing proteins in plants ^51,52^.

### Proximity labelling by MpTACO1 support a role in organelle protein biosynthesis

**Fig. 5:**
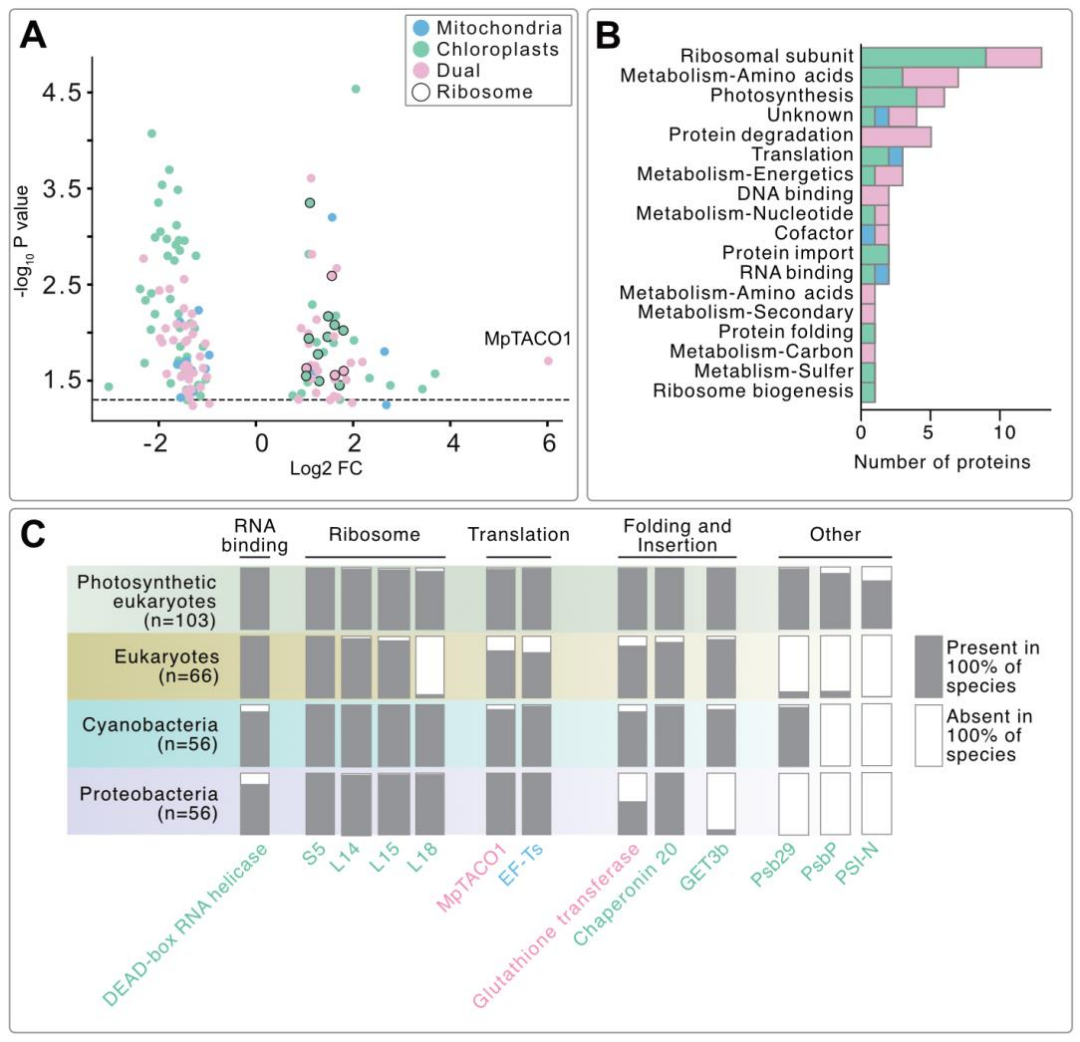
Proximity-labelling by TACO1:miniTURBO in mitochondria and plastids. **(A)** Log_2_ fold change (Log_2_ FC) in quantity of organelle-localized proteins and between plant lines expressing MpTACO1::miniTurbo and WT against −log_10_ P value. Each dot represents a protein localized to mitochondria, plastids or both as per the key. Outlined dots represent ribosomal subunits and dots above the dotted line indicate a significant difference between MpTACO1::miniTurbo and WT lines (Log_2_ FC>1 indicate enrichment; Log_2_ FC<1 indicate depletion). **(B)** Functional categories of organelle-localized proteins enriched in miniTurbo lines. Stacks within each bar represent protein localization as per the key in A. **(C)** Evolutionary conservation of a subset of enriched proteins that are involved in information processing (transcription and translation) and photosynthesis in plastids across eukaryotes bearing plastids, other eukaryotes, cyanobacteria and proteobacteria (n, number of species in each category). Protein names are colour-coded to represent their localization as per the key in A.

To identify potential interaction partners of MpTACO1 and further verify the dual localisation of the protein in the vicinity of organelle ribosomes, we fused the full-length protein to miniTurbo, a modified biotin ligase^53^. We selected for isogenic Marchantia plant lines expressing the construct in the absence of antibiotic selection pressure (Fig. S12A). The fusion to miniTurbo did not affect the plastid targeting of MpTACO1, as evident by immunoblotting from plastids isolated from the two plant lines (Fig. S12B-C). We isolated biotinylated proteins and determined their identity through mass spectrometry. As expected from the dual localization, the biotinylated proteins included 28 dual-, 39 plastid- and seven mitochondrial proteins (Fig. 5A, Table S3). In line with an expected role in protein synthesis, ten of the dual- and twelve plastid-localized proteins were ribosomal subunits, which included S13, S5, S14 (plastid-encoded), L18, and L3; these results are similar to the BioID-tagged human TACO1^54^. Broad functional categories also underscored interactors involved in photosynthesis, energetics, protein folding and degradation (Fig. 5B). The presence of membrane and thylakoid localized proteins with unknown functions, and a homolog of a chloroplast localized GET protein (*Arabidopsis* AT3G10350), places MpTACO1 in the vicinity of membranes, maybe at the site of membrane protein insertion. More than a dozen candidate interaction partners, including RNA binding protein, ribosomal subunits, and the GET3 homolog were found to be highly conserved across bacteria and eukaryotes (Fig. 5C). The proteins identified by proximity labelling confirm the dual-localization of MpTACO1 and support its role in translation in mitochondria and plastids.

## Discussion

We provide evidence for the selection and retention of a single YebC/TACO1 homolog of bacterial origin in algae and plants, which is dual targeted to mitochondria and plastids. Data from bacteria and mitochondria demonstrate the protein acts as an auxiliary protein to their ribosomes and helps to stabilize the PTC during the translation of proline motifs^37–39,41^. Organelle-encoded proteins often nucleate the early assembly of complexes for oxidative phosphorylation and photosynthesis, respectively at the inner mitochondrial or thylakoid membrane. This process requires the coordination of an import machinery, organellar translation, assembly factors, and the co-translational membrane insertion of nascent peptide chains and the addition of metal moieties such as heme or chlorophyll.

Unassembled subunits, in particular those involving metal cofactors, are problematic for mitochondria and plastids, as evident from an elaborated machinery that has evolved for their rapid degradation^55–60^ in a way that resembles the envelope stress response known from bacteria^61^. Organelle translation must hence secure the spatiotemporal translation, folding and correct insertion at their membranes. In this respect, ribosomal stalling by PP motifs could offer a regulatory mechanism. Prolonged stalling, however, is simultaneously problematic and numerous rescue factors such as EF-P, eIF5a, and the ABCF protein family emerged – likely together with the origin of cellular life itself – to deal with motifs that can slow down ribosomes^35^. These factors have been co-evolving to for billions of years with their respective ribosomes in bacteria as well as eukaryotic organelles of bacterial origin. While cytosolic, and mitochondrial translation with respect to proline motifs and the recruitment of YebC/TACO1 proteins has been explored^37–39,41^, this is not yet the case for plastid ribosomes.

Polyproline motifs are a common cause of stalling, especially when preceded by D/A or followed by G/W/N/D ^62^. Among the more than 15,000 plastid genomes we screened, 29.4% of the encoded proteins harbour at least one PP motif. That is less than the 41.7% of cyanobacterial proteins and showcases the different evolutionary forces acting on plastid genomes that reduce the number of GC-rich codons in general^44,45^. The presence of PP motifs encoded by CCN hints at a retention for reasons beyond their ability to adopt unique right- or left-handed type I and II helices^63^. Proline residues present a dilemma for translation at the PTC due to the biochemical nature of the amino acid, and which is likely why in fast growing bacteria the frequency of the amino acid is reduced^35,64^. The high frequency of PP motifs retained across all domains of life, however, is testimony to their benefit or even necessity (e.g. as a helix breaker). An analysis of 43 *Escherichia coli* strains underscores the regulatory function of PP motifs^65^ that also have a tendency to increase in number in more complex eukaryotes^66^. PP motifs encoded on plastid genomes are also found in the vicinity of transmembrane domains or at C-termini (Fig. S3), which could aid (i) their insertion by insertases of the OXA family in both mitochondria and plastids^67,68^ and (ii) steps in the maturation of a protein such as the addition of metal cofactors. Thus, emerging data appears at odds with literature that view PP motifs as a mere inconvenience during translation^35^ or only as helix breakers during protein folding, and neglect their potential to act as indicators for events in translation that require pausing^69–71^. The evolutionary retention of auxiliary proteins shows that ribosomes require them to manage the rate of translation of PP motifs encoded within the mRNA. Even though ribosomes can synthesise peptides encoding PP motifs in the absence of additional factors^72^, the rate can be dramatically slower depending upon the context of the flanking amino acid residues. This points to temporal regulation of nascent chain synthesis, folding and membrane insertion. A broad and global analysis of protein sequence features upstream of polyproline motifs could address this question and might identify different functional categories for the motifs.

Mitochondrial ribosomes require TACO1 for elongation of at least some proteins enriched in PPs, and we show TACO1 to be imported also by Marchantia plastids (Fig. 4). Proximity labelling using a miniTurbo-tagged MpTACO1 (Fig. 5) identified 28 dual targeted proteins, supporting the dual localization of MpTACO1. Enrichment of ribosomal subunits, translation factors, and TACO1 itself, suggest an interactome similar to that previously identified in mitochondria and a similar function of MpTACO1 in plastids; a plastid GET3B homolog^73^ potentially places MpTACO1 in the vicinity of active co-translational membrane insertion sites. Like Marchantia, and the vast majority of plants, maize and Arabidopsis encode a single TACO1 homolog (S1B) and public databases such as TAIR list the protein to be plastid-localised.

Our structure of the first plant TACO1 homolog, in combination with a bioinformatic analysis, underscores a level of conservation that matches that of ribosomal proteins. It speaks for the concatenated evolution of the accessory protein that is recruited to the peptidyl transfer centre (PTC) when both A- and P-sites are occupied by prolyl-tRNAs^38^, ever since its origin in the last common ancestor of bacteria^35^. Accordingly, our modelling shows that chlororibosomes can accommodate both the human and Marchantia TACO1 in the same orientation at the entry site as demonstrated previously for mitoribosomes^43^ (Fig. 3). With respect to our SAXS data, the closed conformation is consistent with the X-ray model and with the cryoEM reconstruction of the human TACO1 that is bound to mitoribosomes^43^. Thus, localisation-, structural-, proximity labelling-, and phylogenomic data point to the plant YebC/TACO1 having a similar function in the plastids of algae and plants as its homolog in the mitochondria of non-photosynthetic eukaryotes^40,43^ and the cytosol of bacteria^38,39^. This extends the range of substrates, whose translation is enhanced by eukaryotic YebC/TACO1 homologs, supporting its importance for the translation of bioenergetic membrane proteins of endosymbiotic organelles.

The single copy conservation of a YebC/TACO1 contrasts with the otherwise observed expansion of organelle-related protein families in land plants^46,74^. Encoding YebC/TACO1 paralogs seems unfavourable, both in pro- and eukaryotes, and one YebC copy was selected against early in the evolution of the first photosynthetic eukaryote – the primal alga – before the diversification into the three main archaeplastidal lineages (Fig. 1A, Fig. S1B). Phylogenomics was not able to resolve which one (Fig. 1C), but we would speculate the cyanobacterial YebC, since the mitochondrial TACO1 was already nuclear-encoded with a functional targeting sequence. Dual targeting to mitochondria and plastids is observed more frequently for proteins of transcription and translation^52^ and thought to require a combination of balanced charges, serine residues and a mitochondrial membrane potential^51,75,76^. Research on dual-targeting outside of *Arabidopsis* is meagre, reporter-based studies are rare and algorithm-based predictions of dual targeting are error-prone^77^. Our growth conditions and detection limits did not capture a localisation shift for the serine-to-alanine mutants, adding further mystery to the role of the significant enrichment of serine residues in pTPs, but the three transcription and translation-associated proteins that we show are dually targeted in the bryophyte in addition to TACO1 (Fig. S11) support the described pattern for *Arabidopsis*^52^ and can help to experimentally explore the extent, evolution, and regulation of dual targeting in plants.

## Conclusion

The bioenergetic membranes of mitochondria and plastids are of bacterial origin. The maturation of their protein complexes requires co-ordination of translation, modification and insertion of a variety of proteins such as COX1 into the inner mitochondrial membrane or D1 (psbA) into the thylakoids of plastids. Both these proteins contain conserved PP motifs that are also frequent across other organelle-encoded proteins, and many of which are evolutionary conserved and known to recruit accessory proteins to the ribosome during translation. YebC/TACO1 is one such protein required by mitochondria and its targeting to plastid suggest a similar role in plastid translation of at least a subset of about two dozen PP-containing proteins underscored here. As for the presence of PPs, we speculate that the retention of PP motifs encoded by GC-rich codons in AT-rich genomes hint at a regulatory potential in translation elongation for nascent chain folding and membrane insertion. While the mechanism of the YebC/TACO1 recruitment to ribosomes remains uncertain, one can synthesize a model covering bacteria, mitochondria and plastids. The PTC site of their ribosomes is constantly probed by YebC/TACO1 proteins that are rapidly changing between conformations. Formation of peptide bonds involving prolines is slower, triggering a conformational shift in domain V of the 23S rRNA (or its mitochondrial equivalent, the 16S rRNA), which recruits YebC/TACO1 that can then interlock in its closed conformation to stabilize the PTC site. We propose the protein family name be changed to Translation Enhancer of (Poly)Prolines (TEPP) proteins based upon the following data: (a) the localisation of TACO1 to plastids suggest a role in translation regulation beyond the mitochondria-encoded COX1; (b) the factors bind primarily rRNA, not mRNA, across different ribosomes; (c) the factors regulate the rate of translation elongation and not activate translation initiation; and (d) the robust structural and functional conservation since YebC origin. Our data hint at a so far untapped feature of plastid biology, which is proline residue translation that requires ancient accessory ribosomal proteins of the TEPP family such as YebC or TACO1.

## Methods

### Comparative genomics

We searched for poly-proline regions (PPs; defined as at least two consecutive prolines in protein sequence) across all chloroplast and mitochondrial genomes available on NCBI-Refseq (accessed in March 2026) using an inhouse python script (‘count_PPs.ipyub’). To search for ribosomal rescue factors across species, we clustered whole proteome models of 169 eukaryotic, 57 proteobacterial and 56 cyanobacterial genomes using OrthoFinder version 2.5.4^78^ into protein orthologues (Table S4) and identified ribosomal rescue factors using *Arabidopsis*, Human or *E. coli* gene IDs of rescue factors reported previously^35^. For generating a phylogenetic tree of TACO1/yebC homologs, we aligned protein sequences of 13 diverse eukaryotes, 7 diverse cyanobacteria and 8 diverse proteobacteria using MAFTT (v7.525)^79^, and removed the N terminal targeting peptide extension (region that did not align with prokaryotic homologs) from the eukaryotic homologs before generating a phylogenetic tree and predicting structures. The phylogenetic tree was generated using IQTree v1.6.12^80^ and structures were predicted using AlphaFold3^47^. The models with highest confidence were used for structural comparison on DALI^81^.

### MpTACO1 expression, purification and crystallisation

A recombinant plasmid containing the MpTACO1 coding sequence (Mp1g23800) was constructed using Gibson assembly. The coding sequence excluding the predicted mitochondrial targeting sequence was amplified from pDONR221_MpTACO1_NoNTS using Mar_For (ACAGAGAACAGATTGGTGGAATGGGGAGACGTTCGGCCAAG) and Mar_Rev primers (ACTCGAGTGCGGCCGCAAGCTTATTACTTTTGATTACAATAGACAGCATCCACA). Polymerase chain reaction (PCR) amplification was performed with the Platinum SuperFi II Green PCR Master Mix (Invitrogen) with 0.5 μM primers and 2 ng of template DNA. The backbone plasmid for the Gibson assembly was pFRGSF01^82^, which encodes for both a hexa-histidine tag and a SUMO tag at the N-terminus. pFRGSF01 was amplified using primers pFR_For (GCTTGCGGCCGCACTCGAGT) and pFR_Rev (TCCACCAATCTGTTCTCTGTGAGTCTCA) with the Platinum SuperFi II Green PCR Master Mix (Invitrogen), 0.5 μM primers, and 10 ng of template DNA. The PCR product was digested with DpnI (Thermo Scientific). Both PCR products were purified using a NucleoSpin Gel and PCR Clean-up kit (Macherey-Nagel). For the Gibson assembly, a NEBuilder HiFi DNA Assembly Cloning Kit (New England BioLabs) was used. The assembly reactions were transformed into XL10 Gold electrocompetent (E. coli cells and incubated overnight at 37 °C on Luria-Bertani agar supplemented with 25 μg mL-1 kanamycin.

For expression, the recombinant plasmid was transformed into E. coli BL21(DE3) and the gene was expressed in autoinduction media (AIM-LB broth base including trace elements, Formedium). Cultures were grown at 37°C with shaking until they reached an optical density (OD600) of 0.6, after which the temperature was reduced to 20°C for 16h. Cells were collected by centrifugation (2719 RCF Rmax, 10 min, 4°C). The cell pellets were resuspended in 50 mL 20 mM Tris-HCl pH 8.0, 150 mM NaCl, 30 mM imidazole pH 8.0 supplemented with 1 tablet of Pierce^™^ Protease Inhibitor Tablets, EDTA-Free (Thermo Scientific) and then lysed using an EmulsiFlex-C3 (Avestin). Cell debris was removed by centrifugation (26712 RCF Rmax, 30 min, 4°C). The clarified lysate was incubated with immobilized metal ion affinity chromatography Sepharose^™^ 6 Fast Flow resin (GE Healthcare) pre-charged with 0.1 M NiSO_4_. The resin was washed with five column volumes of 20 mM Tris-HCl pH 8.0, 150 mM NaCl, and 30 mM imidazole pH 8.0, then packed into a disposable PD-10 gravity column (Cytiva) and washed again with five column volumes of the same buffer. Bound proteins were step-eluted with 20 mM Tris-HCl pH 8.0, 150 mM NaCl, 200 mM imidazole pH 8.0, and collected as 1 mL fractions. The fractions with the highest protein concentration and reasonable purity were combined and diluted with 20 mM Tris-HCl pH 8.0, 50 mM NaCl cleaved with SUMO protease on ice for 1 hour. Further purification on a HisTrap^™^ FF crude 5 mL column and HiTrap^™^ Q HP 1 mL (Cytiva) column in tandem, prior to elution from the The HiTrap^™^ Q column with a linear gradient from 50 mM Tris-HCl pH 8.0 to 100% 50 mM Tris-HCl pH 8.0, 1M NaCl. Finally, the peak fractions were polished on a Superdex^™^ 75 10/300 GL gel filtration column (Cytiva) equilibrated with 50 mM Tris-HCl pH 8.0, 150 mM NaCl.

Crystallisation experiments were conducted in the FINStruct HiLIFE Crystallography Unit at the Institute of Biotechnology, University of Helsinki, using a mosquito^®^ LCP (SPT Labtech). Purified recombinant MpTACO1 was concentrated to 13.7 mg mL^−1^, using Sartorius Vivaspin^®^ 500 Centrifugal Concentrator (10,000 MWCO). Crystallisation was carried out by sitting drop vapor diffusion at 20°C, with 1:1 (v/v) mixtures of protein sample and crystallization solution (100 nL each). The crystallisation plates were imaged using a Rock Imager 182 (Formulatrix). Diffracting plates were obtained after 20 h from the Helsinki cryoscreen (https://www.helsinki.fi/en/infrastructures/integrated-structural-cell-biology/infrastructures/crystallization/standard-screens, accessed 17.3.2025) from a main solution of 0.1M MES, pH 6, 30% (v/v) polyethylene glycol 200, 5% (w/v) polyethylene glycol 3350. Data was collected at beamline ID30A-3 (ESRF, Grenoble, France) at a wavelength of 0.9677 Å. The X-ray diffraction data were auto-processed with the GrenADES parallelproc pipeline. The Alphafold3^47^ model without the predicted MTS was for molecular replacement using Phaser^83^ as implemented in the CCP4 Cloud ^84^. The structure was initially refined using *REFMAC5*^85^ and ModelCraft^86^, followed by several rounds of manual building in *Coot*^87^ with further refinement and validation in PHENIX^88^. Figures were prepared in UCSF ChimeraX^89^

### Small angle X ray scattering (SAXS) analyses on MpTACO1

We collected the SAXS data on our Xeuss 2.0 Q-Xoom system from Xenocs, equipped with a PILATUS 3 R 300K detector (Dectris) and a GENIX 3D CU Ultra Low Divergence x-ray beam delivery system. The chosen sample to detector distance for the experiment was 0.55 m, results in an achievable q-range of 0.05 – 5.5 nm^−1^. The measurement was performed at 15°C with a protein concentration of 5.44 mg/ml. The sample was injected in the Low Noise Flow Cell (Xenocs) via autosampler. We collect 36 frames for buffer and protein with an exposer time of ten minutes/frame and scaled the data to absolute intensity against water. Data frames were checked for radiation damage and averaged, leading to buffer subtracted protein data. All used programs for data processing were part of the ATSAS Software package (Version 3.0.5)^90^. Primary data reduction was performed with the program PRIMUS^91^. With the Guinier approximation^92^, we determine the forward scattering I(0) and the radius of gyration $\left(R_{g}\right)$. The program GNOM^93^ was used to estimate the maximum particle dimension $\left(D_{\max}\right)$ with the pair-distribution function p(r). We compared the solved crystal structure with the experimental data using CRYSOL^94^. A Model was created with AlphaFold3^47^ to complete the crystal structure. The helical N- terminal part of the model (domain 1) was set as flexible and remodeled with an Ensemble Optimization Method (EOM)^95,96^.

#### Ribosome-TACO1 modelling

The human mitoribosome-associated TACO1 atomic model (PDB: 9OLF) was fitted to a *Spinacia oleracea* chlororibosome model (wwPDB ID: 5MMM) in UCSF ChimeraX v1.9 using the Matchmaker ChimeraX ^89^. TACO1 from 9OLF was superimposed onto the 5MMM model and respective map. The MpTACO1 crystal structure was similarly fitted to the human TACO1 model with Matchmaker and imaged with the 5MMM model.

### Plant transfectant generation and growth conditions

All transfectants in this study were generated in ecotype Takaragaire-1 (Tak-1) of the liverwort *Marchantia polymorpha*. The wild type gemmae were grown under continuous light (70 μmol m^−2^ s^−1^) on half-strength Gamborg B5 vitamin agar-plates (GVA).

We amplified the genes of interest from *Marchantia,* Mp*TACO1* (Mp1g23800 alias Mapoly0061s0140), aspartyl/glutamyl amidotransferase subunit B (Mp1g29210 alias Mapoly0107s0036), lysyl-tRNA-synthetase (Mp7g09810 alias Mapoly0003s0001), seryl-tRNA-synthetase (Mp6g09660 alias Mapoly0016s0010), LHCA3/LHCII Chla/b binding protein 3 (Mp7g08940 alias Mapoly0068s0047) and MME1 (Mp4g02270 alias Mapoly0080s0072) from the cDNA of 2 week old thalli and cloned into *Gateway* (Invitrogen) entry vector pDONR221^™^ (Invitrogen) by BP reactions set as per manufacturer’s protocol. Substitutions of interest were made into the wild type Mp*TACO1* using PCR-mutagenesis. All constructs were transferred N-terminal of Citrine into pMpGWB106 ^97^ via *Gateway* cloning by LR reactions set as per manufacturer’s protocol.

We integrated the localization vectors with the genes of interest into Marchantia genome via *Agrobacterium* mediated transfections previously optimized ^51,98^. Briefly, the vectors were transfected to electrocompetent *Agrobacterium tumefaciens* (GV3101 lacking pSOUP) by electroporation (Bio-Rad GenePulser Xcell, 1.44 kV) and *Agrobacterium* transfectants were incubated with explants from two-week-old thalli after removing their epical notches and two days of recovery. Transfectants were then selected onto Hygromycin (10 μg/mL) and Cefotaxime (100 μg/mL) and further select for successful reporter expression at gemmae under the microscope. Expression positive gemmae were further propagated and used for the localization experiments after growing for up to three generations by serial passages from the gemma stage.

### Sample preparation for subcellular localization

Localization experiments were conducted in gemmae, 2-day old thalli or protoplasts isolated from 4–7 days (until otherwise stated) old thalli of isogenic transfectant lines. Gemmae or thalli were placed on a glass slide with 30–50μL water and covered with a cover-slip. Protoplasts were isolated as previously described^51^, from the thalli after incubation with 10mL of 8% Mannitol, 6.3 g/L Gamborg B5 Vitamins (MGV) with 15mg/ml Cellulase and 5 mg/mL Macroenzyme R10 and filtration with a 50μm strainer. After two washes with MGV (at 300g, 3min), the protoplasts were used for mitochondrial staining by MitoTracker^™^ Red CMXRos (following the manufacturer’s protocol).

### Microscopy

Microscopy was carried out as described previously ^51^. Briefly, protoplasts were imaged using Nikon eclipse Ti imaging platform (illuminated with Nikon Intensilight C-HGFIE for fluorescence, connected to the camera via Nikon Digital Sight DS-U3 and to the PC via NIS-Elements Basic Research version 4.30) for visualizing chloroplast autofluorescence (480/590 nm, exposure time 20–80ms), Citrine (470/520 nm, exposure time up to 300ms) and mitochondria (540/605, exposure time up to 300ms). 2-day old thallus (in Fig. 3A) were imaged on a Leica TCS SP8 platform (Leica, Wetzlar, Germany) with an HC PL APO CS2 93× glycerol objective (NA 1.3, Leica), connected to a PC via LAS-X (Leica) and deconvoluted using the Lightning adaptive deconvolution module; plastid autofluorescence was filtered out while scanning for Citrine signal using time gating^99^. The collages were prepared in ImageJ (Fiji). Co-localisation analyses were conducted using BIOP JACoP plugin.

### Plastid isolation

Plastids were isolated following the lysis of 2-week-old thalli, in presence of 5–10 mL isolation buffer (50mM HEPES KOH pH 7.5, 0.33 M sorbitol, 1mM MgCl_2_, 1mM MnCl_2_, 2mM EDTA pH 8, 0.5% w/v BSA, 0.5% w/v PVP40, and freshly added 5mM ascorbate), the lysate was filtered through a Mira cloth and centrifuged at 1000g, 10min. The pellet was resuspended in IB and loaded onto a discontinuous gradient of 70–30% Percoll. Followed by density gradient centrifugation at 4300g, intact plastids were harvested from the 70–30 layer, washed with wash buffer (WB; IB without BSA and PVP40) three times by centrifugation for 10min at 3000g, 200og and 1000g. Plastids were pulverized in presence of liquid nitrogen and crude plastid lysate was used for immunoblotting.

### Interaction partners of MpTACO1

We investigated interaction partners of MpTACO1 using mini-turbo based method optimized previously for *Marchantia*^53^. Briefly, we fused the full length MpTACO1 at the N terminal of miniTurbo protein onto vector pMKMM2 via *Gateway* cloning by LR reactions set as per manufacturer’s protocol. The final constructs were transfected to Marchantia as per above and transfectants were selected on 0.5μM Chlorsulfuron for two generations and used for the biotinylation assay in the third generation. For biotinylation, gemmae (10–12 gemmae per plate, four plate per replicate) were grown for 10 days, and thalli were soaked into 600μM biotin (ca. 2–3 thalli per 10mL biotin solution in water) subjected to 5 min of vacuum infiltration and incubated further for 20 hours. All thalli from a single replicate were pooled and washed three times with 50ml water and tapped briefly on tissue paper for the removal of water prior to lysis. Dried thalli were pulverized in liquid nitrogen, the power resuspended in SDT buffer (100 mM Tris, pH 7.5, 4% SDS, and 0.1 M DTT), incubated at 45C for 15min for resuspension. Lysate was cleared by two rounds of centrifugation at 10,000g 15min, and used for protein isolation. To isolate protein, 666μL methanol was mixed well by vortexing with 500μL lysate, mixed further with 166μL chloroform and 300μL water. After centrifuge at 10,000g 10min the upper and lower liquid layers were pipetted out to retain only the middle proteinaceous middle layer in the tube. This layer was washed with 600μL methanol by mixing well with pipetting and sonication in ultrasonic bath for 10min. This was centrifuged at 10,000g, 10min and washed again. Methanol was removed by decanting after the second wash and by leaving the tube upside down for 1–2minutes. The pellet was resuspended in 500μL SDT at 37°C for 10min while shaking at 1000rpm, sonicated in ultrasonic bath for 10min, and again incubation at 37°C for 10min while shaking at 1000rpm. The resuspended proteins were used for affinity pulldown.

100μL of 50% Streptavidin agarose bead slurry was mixed with 5mL binding buffer (0.1M phosphate, 0.15M NaCl, 0.5% SDS, pH 7.2), mixed by inverting 5–10 times and centrifuged for 2min at 3500g at room temperature (this step was repeated three times). 500μL of isolated protein was added to 3.5mL of wash buffer 2 (0.1M phosphate, 0.15M NaCl, pH7.2) and transferred to streptavidin beads and mixed by inverting 5–10 times. After 18h incubation at 22°C (gentle rocking) beads were centrifuged at 3500g 3min and removal of as much supernatant as possible. The beads were washed by wash buffer 1 (0.1M phosphate,0.15M NaCl, 2% SDS, pH 7.2) four times. The beads were incubated in Laemmli buffer, centrifuged and supernatant was resolved on SDS-PAGE and individual gel pieces were reduced with 50 μl 10 mM DTT, alkylated with 50 μl 50 mM iodoacetamide and digested with 200ng trypsin in 100 mM ammonium bicarbonate. Peptides were subjected to liquid chromatography after resolving them in 15 μl 0.1% trifluoroacetic acid. LC-MS was conducted on QExactive Plus (Thermo Scientific) connected to an Ultimate 3000 Rapid Separation liquid chromatography system (Thermo Scientific), equipped with an Aurora Ultimate column (75 μm inner diameter, 25 cm length, 1.7 μm particle size, IonOpticks) as the analytical separation column and an Acclaim PepMap 100 C18 column (75 μm inner diameter, 2 cm length, 3 μm particle size, Thermo Fisher Scientific) as the trap column (LC gradient length of 180 minutes). The mass spectrometer was operated in positive mode and coupled to a nano-electrospray ionization source (capillary temperature 250 °C, source voltage to 1.7 kV). Survey scans were acquired over a mass range of 350–2000 m/z at a resolution of 140,000. The 10 most intense peptide ions were isolated and fragmented by high-energy collision dissociation (HCD). Two replicates per independent MpTACO1::miniTurbo transfectant lines (Bio2 and Bio8) and two replicates of the WT were subjected to biotinylation treatment and mass spectrometry. The protein quantities of each protein from the four replicates from transfectant lines were compared with that in the WT to get log_2_FC ratio of each protein and proteins with absolute log_2_FC above 1 (p value <0.05, t-test) in at least three out of four replicates, were retained as candidate interaction partners.

## Supplementary Material

Supplement 1

## Data Availability

Supplementary figures and tables 1–2 are available with this submission. Additional data are available on Zenedo (https://zenodo.org/records/20426666), which includes, but is not limited to, the supplementary data, source data for the main and supplementary figures, supplementary tables, and in-house scripts. The X-ray crystallographic data are deposited with the wwPDB ID: 9SCZ and the SAXS data to the Small Angle Scattering Biological Data Bank (SASBDB)^100^ with the accession code SASDYA7.

## Highlights

- Dozens of GC-rich polyproline (PP) encoding regions are retained by AT-rich genomes
- PP motif conservation hints at regulatory mechanisms and required translation pauses
- Chloroplast targeting of a (mitochondrial) translation enhancer of PP motifs
- MpTACO1 structure at 2.34 Å resolution demonstrates its high level of conservation

## References

[1] Mills D.B., Boyle R.A., Daines S.J., Sperling E.A., Pisani D., Donoghue P.C.J., and Lenton T.M. (2022). Eukaryogenesis and oxygen in Earth history. Nat. Ecol. Evol. 6, 520–532. 10.1038/s41559-022-01733-y. 35449457

[2] Raval P.K., Garg S.G., and Gould S.B. (2022). Endosymbiotic selective pressure at the origin of eukaryotic cell biology. Elife 11, 1–23. 10.7554/ELIFE.81033. PMC9648965 36355038

[3] Martin W.F., Garg S., and Zimorski V. (2015). Endosymbiotic theories for eukaryote origin. Philosophical Transactions of the Royal Society B: Biological Sciences 370. 10.1098/rstb.2014.0330. PMC4571569 26323761

[4] Bowles A.M.C., Williamson C.J., Williams T.A., Lenton T.M., and Donoghue P.C.J. (2023). The origin and early evolution of plants. Preprint at Elsevier Ltd, 10.1016/j.tplants.2022.09.009 https://doi.org/10.1016/j.tplants.2022.09.009. 36328872

[5] Gould S.B., Waller R.F., and McFadden G.I. (2008). Plastid evolution. Annu. Rev. Plant Biol. 59, 491–517. 10.1146/annurev.arplant.59.032607.092915. 18315522

[6] Archibald J.M. (2015). Endosymbiosis and eukaryotic cell evolution. Current Biology 25, R911–R921. 10.1016/j.cub.2015.07.055. 26439354

[7] Timmis J.N., Ayliff M.A., Huang C.Y., and Martin W. (2004). Endosymbiotic gene transfer: Organelle genomes forge eukaryotic chromosomes. Nat. Rev. Genet. 5, 123–135. 10.1038/nrg1271. 14735123

[8] Kelly S. (2021). The economics of organellar gene loss and endosymbiotic gene transfer. Genome Biol. 22. 10.1186/s13059-021-02567-w. PMC8686548 34930424

[9] Martin W., and Herrmann R.G. (1998). Gene Transfer from Organelles to the Nucleus: How Much, What Happens, and Why? Plant Physiol. 118, 9–17. 10.1104/pp.118.1.9. 9733521 PMC1539188

[10] Kleine T., Maier U.G., and Leister D. (2009). DNA transfer from organelles to the nucleus: The idiosyncratic genetics of endosymbiosis. Annu. Rev. Plant Biol. 60, 115–138. 10.1146/annurev.arplant.043008.092119. 19014347

[11] Schleiff E., and Becker T. (2011). Common ground for protein translocation: Access control for mitochondria and chloroplasts. Nat. Rev. Mol. Cell Biol. 12, 48–59. 10.1038/nrm3027. 21139638

[12] den Brave F., Schulte U., Fakler B., Pfanner N., and Becker T. (2024). Mitochondrial complexome and import network. Trends Cell Biol. 34, 578–594. 10.1016/j.tcb.2023.10.004. 37914576

[13] Wiedemann N., and Pfanner N. (2017). Mitochondrial Machineries for Protein Import and Assembly. Annu. Rev. Biochem. 86, 685–714. 10.1146/annurev. 28301740

[14] Jarvis P., and López-Juez E. (2013). Biogenesis and homeostasis of chloroplasts and other plastids. Nat. Rev. Mol. Cell Biol. 14, 787–802. 10.1038/nrm3702. 24263360

[15] Allen J.F., Horner D.S., Cavalier-Smith T., Willison K., Leaver C.J., and Martin W. (2003). The function of genomes in bioenergetic organelles. In Philosophical Transactions of the Royal Society B: Biological Sciences (Royal Society), pp. 19–38. 10.1098/rstb.2002.1191. PMC1693096 12594916

[16] Maier U.G., Zauner S., Woehle C., Bolte K., Hempel F., Allen J.F., and Martin W.F. (2013). Massively convergent evolution for ribosomal protein gene content in plastid and mitochondrial genomes. Genome Biol. Evol. 5, 2318–2329. 10.1093/gbe/evt181. 24259312 PMC3879969

[17] Gray M.W., Lang B.F., Cedergren R., Golding G.B., Lemieux C., Sankoff D., Turmel M., Brossard N., Delage E., Littlejohn T.G., (1998). Genome structure and gene content in protist mitochondrial DNAs. Nucleic Acids Res. 26. 10.1093/nar/26.4.865 PMC147373 9461442

[18] Giannakis K., Arrowsmith S.J., Richards L., Gasparini S., Chustecki J.M., Røyrvik E.C., and Johnston I.G. (2022). Evolutionary inference across eukaryotes identifies universal features shaping organelle gene retention. Cell Syst. 13, 874–884.e5. 10.1016/j.cels.2022.08.007. 36115336

[19] Björkholm P., Ernst A.M., Hagström E., and Andersson S.G.E. (2017). Why mitochondria need a genome revisited. FEBS Lett. 591, 65–75. 10.1002/1873-3468.12510. 27928819

[20] Björkholm P., Harish A., Hagström E., Ernst A.M., and Andersson S.G.E. (2015). Mitochondrial genomes are retained by selective constraints on protein targeting. Proc. Natl. Acad. Sci. U. S. A. 112, 10154–10161. 10.1073/pnas.1421372112. 26195779 PMC4547212

[21] von Heijne G. (1986). Why mitochondria need a genome. FEBS Lett. 198, 1–4. 10.1016/0014-5793(86)81172-3. 3514271

[22] Allen J.F. (2015). Why chloroplasts and mitochondria retain their own genomes and genetic systems: Colocation for redox regulation of gene expression. Proc. Natl. Acad. Sci. U. S. A. 112, 10231–10238. 10.1073/pnas.1500012112. 26286985 PMC4547249

[23] Race H.L., Herrmann R.G., and Martin W. (1999). Why have organelles retained genomes ? Trends Genet. 15, 364–370. 10.1016/s0168-9525(99)01766-7. 10461205

[24] Allen J.F., and Martin W.F. (2016). Why have organelles retained genomes? Cell Syst. 2, 70–72. 10.1016/j.cels.2016.02.007. 27135161

[25] Ueda M., Nishikawa T., Fujimoto M., Takanashi H., Arimura S.I., Tsutsumi N., and Kadowaki K.I. (2008). Substitution of the gene for chloroplast RPS16 was assisted by generation of a dual targeting signal. Mol. Biol. Evol. 25, 1566–1575. 10.1093/molbev/msn102. 18453549

[26] Kubo N., and Arimura S.I. (2010). Discovery of the rpl10 gene in diverse plant mitochondrial genomes and its probable replacement by the nuclear gene for chloroplast RPL10 in two lineages of angiosperms. DNA Research 17, 1–9. 10.1093/dnares/dsp024. 19934175 PMC2818186

[27] Kizmaz B., and Herrmann J.M. (2023). Membrane insertases at a glance. J. Cell Sci. 136. 10.1242/jcs.261219. 37417332

[28] Hegde R.S., and Keenan R.J. (2024). A unifying model for membrane protein biogenesis. Nat. Struct. Mol. Biol. 31, 1009–1017. 10.1038/s41594-024-01296-5. 38811793 PMC7616256

[29] Gawroński P., Jensen P.E., Karpiński S., Leister D., and Scharff L.B. (2018). Pausing of chloroplast ribosomes is induced by multiple features and is linked to the assembly of photosynthetic complexes. Plant Physiol. 176, 2557–2569. 10.1104/pp.17.01564. 29298822 PMC5841727

[30] Wakigawa T., Mito M., Ando Y., Yamashiro H., Tomuro K., Tani H., Tomizawa K., Chujo T., Nagao A., Suzuki T., (2025). Monitoring the complexity and dynamics of mitochondrial translation. Mol. Cell 85, 4279–4297.e8. 10.1016/j.molcel.2025.10.022. 41232526

[31] Woolstenhulme C.J., Parajuli S., Healey D.W., Valverde D.P., Petersen E.N., Starosta A.L., Guydosh N.R., Johnson W.E., Wilson D.N., and Buskirk A.R. (2013). Nascent peptides that block protein synthesis in bacteria. Proc. Natl. Acad. Sci. U. S. A. 110. 10.1073/pnas.1219536110. PMC3593848 23431150

[32] Sabi R., and Tuller T. (2015). A comparative genomics study on the effect of individual amino acids on ribosome stalling. BMC Genomics 16, 1–12. 10.1186/1471-2164-16-S10-S5. 26449596 PMC4602185

[33] Nadler F., Lavdovskaia E., and Richter-Dennerlein R. (2022). Maintaining mitochondrial ribosome function: The role of ribosome rescue and recycling factors. RNA Biol. 19, 117–131. 10.1080/15476286.2021.2015561. 34923906 PMC8786322

[34] Chang W.D., and Choe Y.J. (2026). Quality control and signaling pathways at stalled ribosomes. Exp. Mol. Med. 10.1038/s12276-025-01623-w. PMC12868832 41535548

[35] Lassak J., Stritzel J., Schlundt A., and Brewer T. (2026). Constant trouble with prolines-navigating a global translation dilemma. Nucleic Acids Res. 54. 10.1093/nar/gkaf1420. PMC12856214 41614299

[36] Melnikov S., Mailliot J., Rigger L., Neuner S., Shin B., Yusupova G., Dever T.E., Micura R., and Yusupov M. (2016). Molecular insights into protein synthesis with proline residues. EMBO Rep. 17, 1776–1784. 10.15252/embr.201642943. 27827794 PMC5283605

[37] Hong H.R., Prince C.R., Wu L., Lin I.N., Callan K., and Feaga H.A. (2025). YebC2 resolves ribosome stalling and increases fitness of cells lacking EF-P and the ABCF ATPase YfmR. PLoS Genet. 21. 10.1371/journal.pgen.1011633. PMC11990639 40215226

[38] Ignatov D., Shanmuganathan V., Ahmed-Begrich R., Alagesan K., Hahnke K., Wang C., Krause K., Cornejo F.A., Funke K., Erhardt M., (2025). RNA-binding protein YebC enhances translation of proline-rich amino acid stretches in bacteria. Nature Communications 16, 6262. 10.1038/s41467-025-60687-4. PMC12234827 40624002

[39] Brischigliaro M., Krüger A., Moran J.C., Antonicka H., Ahn A., Shoubridge E.A., Rorbach J., and Barrientos A. (2024). The human mitochondrial translation factor TACO1 alleviates mitoribosome stalling at polyproline stretches. Nucleic Acids Res. 52, 9710–9726. 10.1093/nar/gkae645. 39036954 PMC11381339

[40] Richman T.R., Spahr H., Ermer J.A., Davies S.M.K., Viola H.M., Bates K.A., Papadimitriou J., Hool L.C., Rodger J., Larsson N.G., (2016). Loss of the RNA-binding protein TACO1 causes late-onset mitochondrial dysfunction in mice. Nat. Commun. 7. 10.1038/ncomms11884. PMC4915168 27319982

[41] Weraarpachai W., Antonicka H., Sasarman F., Seeger J., Schrank B., Kolesar J.E., Lochmüller H., Chevrette M., Kaufman B.A., Horvath R., (2009). Mutation in TACO1, encoding a translational activator of COX I, results in cytochrome c oxidase deficiency and late-onset Leigh syndrome. Nat. Genet. 41, 833–837. 10.1038/ng.390. 19503089

[42] Seeger J., Schrank B., Pyle A., Stucka R., Lörcher U., Müller-Ziermann S., Abicht A., Czermin B., Holinski-Feder E., Lochmüller H., (2010). Clinical and neuropathological findings in patients with TACO1 mutations. Neuromuscular Disorders 20, 720–724. 10.1016/j.nmd.2010.06.017. 20727754

[43] Wang S., Brischigliaro M., Zhang Y., Wu C., Zheng W., Barrientos A., and Xiong Y. (2026). Structural basis of TACO1-mediated efficient mitochondrial translation. Nat. Commun. 17, 2521. 10.1038/s41467-026-69156-y. 41663403 PMC12996406

[44] Lynch M., Koskella B, and Schaack S. (2006). Mutation pressure and the evolution of organelle genomic architecture. Science (1979). 311, 1727–1730. 16556832 10.1126/science.1118884

[45] Smith D.R. (2012). Updating our view of organelle genome nucleotide landscape. Front. Genet. 3. 10.3389/fgene.2012.00175. PMC3438683 22973299

[46] Raval P.K., MacLeod A.I., and Gould S.B. (2024). A molecular atlas of plastid and mitochondrial proteins reveals organellar remodeling during plant evolutionary transitions from algae to angiosperms. PLoS Biol. 22. 10.1371/journal.pbio.3002608. PMC11135702 38713727

[47] Abramson J., Adler J., Dunger J., Evans R., Green T., Pritzel A., Ronneberger O., Willmore L., Ballard A.J., Bambrick J., (2024). Accurate structure prediction of biomolecular interactions with AlphaFold 3. Nature 630, 493–500. 10.1038/s41586-024-07487-w. 38718835 PMC11168924

[48] Shin D.H., Yokota H., Rosalind K., and Sung-Hou K. (2002). Crystal structure of conserved hypothetical protein Aq1575 from Aquifex aeolicus. Proceedings of the National Academy of Sciences 99, 7980–7985. 10.1073/pnas.132241399 PMC123006 12060744

[49] Franklin M.C., Cheung J., Rudolph M.J., Burshteyn F., Cassidy M., Gary E., Hillerich B., Yao Z.K., Carlier P.R., Totrov M., (2015). Structural genomics for drug design against the pathogen Coxiella burnetii. Proteins: Structure, Function and Bioinformatics 83, 2124–2136. 10.1002/prot.24841. 26033498

[50] Bieri P., Leibundgut M., Saurer M., Boehringer D., and Ban N. (2017). The complete structure of the chloroplast 70S ribosome in complex with translation factor pY. EMBO J. 36, 475–486. 10.15252/embj.201695959. 28007896 PMC5694952

[51] Raval P.K., García C.G., Sanchez M.-D.S., and Gould S.B. (2025). Evolutionary refinement of mitochondrial and plastid targeting sequences coincides with the late diversification of land plants. Mol. Biol. Evol. 10.1093/molbev/msaf240. PMC12531127 40986664

[52] Carrie C., and Small I. (2013). A reevaluation of dual-targeting of proteins to mitochondria and chloroplasts. Biochim. Biophys. Acta Mol. Cell Res. 1833, 253–259. 10.1016/j.bbamcr.2012.05.029. 22683762

[53] Melkonian K., Stolze S.C., Harzen A., and Nakagami H. (2022). miniTurbo-based interactomics of two plasma membrane-localized SNARE proteins in Marchantia polymorpha. New Phytologist 235, 786–800. 10.1111/nph.18151. 35396742

[54] Antonicka H., Lin Z.Y., Janer A., Aaltonen M.J., Weraarpachai W., Gingras A.C., and Shoubridge E.A. (2020). A High-Density Human Mitochondrial Proximity Interaction Network. Cell Metab. 32, 479–497.e9. 10.1016/j.cmet.2020.07.017. 32877691

[55] Kohler A., Carlström A., Nolte H., Kohler V., Jung S. jun, Sridhara S., Tatsuta T., Berndtsson J., Langer T., and Ott M. (2023). Early fate decision for mitochondrially encoded proteins by a molecular triage. Mol. Cell 83, 3470–3484.e8. 10.1016/j.molcel.2023.09.001. 37751741

[56] Ghandour R., Gao Y., Ruf S., Bock R., and Zoschke R. (2025). Assembly-dependent translational feedback regulation of photosynthetic proteins in land plants. Nat. Plants 11, 1920–1938. 10.1038/s41477-025-02074-x. 40825854 PMC12449265

[57] Imami K., Selbach M., and Ishihama Y. (2023). Monitoring mitochondrial translation by pulse SILAC. Journal of Biological Chemistry 299. 10.1016/j.jbc.2022.102865. PMC9922817 36603763

[58] Bogenhagen D.F., and Haley J.D. (2020). Pulse-chase SILAC- based analyses reveal selective oversynthesis and rapid turnover of mitochondrial protein components of respiratory complexes. Journal of Biological Chemistry 295, 2544–2554. 10.1074/jbc.RA119.011791. 31974161 PMC7049976

[59] Battersby B.J., Richter U., and Safronov O. (2019). Mitochondrial Nascent Chain Quality Control Determines Organelle Form and Function. ACS Chem. Biol. 14, 2396–2405. 10.1021/acschembio.9b00518. 31498990

[60] Koludarova L., and Battersby B.J. (2024). Mitochondrial protein synthesis quality control. Hum. Mol. Genet. 33, R53–R60. 10.1093/hmg/ddae012. 38280230 PMC11112378

[61] Mitchell A.M., and Silhavy T.J. (2019). Envelope stress responses: balancing damage repair and toxicity. Nat. Rev. Microbiol. 17, 417–428. 10.1038/s41579-019-0199-0. 31150012 PMC6596312

[62] Rodnina M. V. (2016). The ribosome in action: Tuning of translational efficiency and protein folding. Protein Science 25, 1390–1406. 10.1002/pro.2950. 27198711 PMC4972197

[63] Umumararungu T., Gahamanyi N., Mukiza J., Habarurema G., Katandula J., Rugamba A., and Kagisha V. (2024). Proline, a unique amino acid whose polymer, polyproline II helix, and its analogues are involved in many biological processes: a review. Amino Acids 56. 10.1007/s00726-024-03410-9. PMC11345334 39182198

[64] Brewer T.E., and Wagner A. (2022). Translation stalling proline motifs are enriched in slow-growing, thermophilic, and multicellular bacteria. ISME Journal 16, 1065–1073. 10.1038/s41396-021-01154-y. 34824398 PMC8941084

[65] Qi F., Motz M., Jung K., Lassak J., and Frishman D. (2018). Evolutionary analysis of polyproline motifs in Escherichia coli reveals their regulatory role in translation. PLoS Comput. Biol. 14. 10.1371/journal.pcbi.1005987. PMC5811046 29389943

[66] Mandal A., Mandal S., and Park M.H. (2014). Genome-wide analyses and functional classification of proline repeat-rich proteins: Potential role of eIF5A in eukaryotic evolution. PLoS One 9. 10.1371/journal.pone.0111800. PMC4218817 25364902

[67] Gerdes L., Bals T., Klostermann E., Karl M., Philippar K., Hünken M., Soll J., and Schünemann D. (2006). A second thylakoid membrane-localized Alb3/OxaI/YidC homologue is involved in proper chloroplast biogenesis in Arabidopsis thaliana. Journal of Biological Chemistry 281, 16632–16642. 10.1074/jbc.M513623200. 16595657

[68] Itoh Y., Andréll J., Choi A., Richter U., Maiti P., Best R.B., Barrientos A., Battersby B.J., and Amunts A. (2021). Mechanism of membrane-tethered mitochondrial protein synthesis. Science (1979). 371, 846–849. 10.1126/science.abe0763. 33602856 PMC7610362

[69] Shingú-Vázquez M., Camacho-Villasana Y., Sandoval-Romero L., Butler C.A., Fox T.D., and Pérez-Martínez X. (2010). The carboxyl-terminal end of Cox1 is required for feedback assembly regulation of Cox1 synthesis in Saccharomyces cerevisiae mitochondria. Journal of Biological Chemistry 285, 34382–34389. 10.1074/jbc.M110.161976. 20807763 PMC2966052

[70] Collart M.A., and Weiss B. (2021). Ribosome pausing, a dangerous necessity for co-translational events. Nucleic Acids Res. 48, 1043–1055. 10.1093/NAR/GKZ763. PMC7026645 31598688

[71] Meier S., Neupert W., and Herrmann J.M. (2005). Proline residues of transmembrane domains determine the sorting of inner membrane proteins in mitochondria. J. Cell Biol. 170, 881–888. 10.1083/jcb.200505126. 16157698 PMC2171449

[72] Doerfel L.K., Wohlgemuth I., Kothe C., Peske F., Urlaub H., and Rodnina M. V. (2013). EF-P Is Essential for Rapid Synthesis of Proteins Containing Consecutive Proline Residues. Science (1979). 339, 85–88. 10.1126/science.1229017. 23239624

[73] Bodensohn U.S., Dünschede B., Kuhlmann C., Kumari K., Ladig R., Grefen C., Schleiff E., Fernandez D., and Schünemann D. (2025). GET3B is involved in chloroplast biogenesis and interacts with the thylakoidal ALB3 and ALB4 insertases. Plant Cell Rep. 44. 10.1007/s00299-025-03500-2. PMC12040988 40299103

[74] Harris B.J., Clark J.W., Schrempf D., Szöllősi G.J., Donoghue P.C.J., Hetherington A.M., and Williams T.A. (2022). Divergent evolutionary trajectories of bryophytes and tracheophytes from a complex common ancestor of land plants. Nat. Ecol. Evol. 6, 1634–1643. 10.1038/s41559-022-01885-x. 36175544 PMC9630106

[75] Law Y.S., Ngan L., Yan J., Kwok L.Y., Sun Y., Cheng S., Schwenkert S., and Lim B.L. (2018). Multiple kinases can phosphorylate the N-terminal sequences of mitochondrial proteins in Arabidopsis thaliana. Front. Plant Sci. 9, 1–14. 10.3389/fpls.2018.00982. 30042778 PMC6048449

[76] Garg S.G., and Gould S.B. (2016). The Role of Charge in Protein Targeting Evolution. Trends Cell Biol. 26, 894–905. 10.1016/j.tcb.2016.07.001. 27524662

[77] Gould S.B., Magiera J., García García C., and Raval P.K. (2024). Reliability of plastid and mitochondrial localisation prediction declines rapidly with the evolutionary distance to the training set increasing. PLoS Comput. Biol. 20, e1012575. 10.1371/journal.pcbi.1012575. PMC11581415 39527633

[78] Emms D.M., and Kelly S. (2019). OrthoFinder: Phylogenetic orthology inference for comparative genomics. Genome Biol. 20, 1–14. 10.1186/s13059-019-1832-y. 31727128 PMC6857279

[79] Madeira F., Madhusoodanan N., Lee J., Eusebi A., Niewielska A., Tivey A.R.N., Lopez R., and Butcher S. (2024). The EMBL-EBI Job Dispatcher sequence analysis tools framework in 2024. Nucleic Acids Res. 52, W521–W525. 10.1093/nar/gkae241. 38597606 PMC11223882

[80] Nguyen L.T., Schmidt H.A., Von Haeseler A., and Minh B.Q. (2015). IQ-TREE: A fast and effective stochastic algorithm for estimating maximum-likelihood phylogenies. Mol. Biol. Evol. 32, 268–274. 10.1093/molbev/msu300. 25371430 PMC4271533

[81] Holm L., Laiho A., Törönen P., and Salgado M. (2023). DALI shines a light on remote homologs: One hundred discoveries. Protein Science 32. 10.1002/pro.4519. PMC9793968 36419248

[82] Guerrero F., Ciragan A., and Iwaï H. (2015). Tandem SUMO fusion vectors for improving soluble protein expression and purification. Protein Expr. Purif. 116, 42–49. 10.1016/j.pep.2015.08.019. 26297996

[83] McCoy A.J., Grosse-Kunstleve R.W., Adams P.D., Winn M.D., Storoni L.C., and Read R.J. (2007). Phaser crystallographic software. J. Appl. Crystallogr. 40, 658–674. 10.1107/S0021889807021206. 19461840 PMC2483472

[84] Krissinel E., Uski V., Lebedev A., Winn M., and Ballard C. (2018). Distributed computing for macromolecular crystallography. Acta Crystallogr. D Struct. Biol. 74, 143–151. 10.1107/S2059798317014565. 29533240 PMC5947778

[85] Kovalevskiy O., Nicholls R.A., Long F., Carlon A., and Murshudov G.N. (2018). Overview of refinement procedures within REFMAC 5: Utilizing data from different sources. Acta Crystallogr. D Struct. Biol. 74, 215–227. 10.1107/S2059798318000979. 29533229 PMC5947762

[86] Bond P.S., Cowtan K.D., and Nicholls R. (2022). ModelCraft: An advanced automated model-building pipeline using Buccaneer. Acta Crystallogr. D Struct. Biol. 78, 1090–1098. 10.1107/S2059798322007732. 36048149 PMC9435595

[87] Emsley P., and Cowtan K. (2004). Coot: Model-building tools for molecular graphics. Acta Crystallogr. D Biol. Crystallogr. 60, 2126–2132. 10.1107/S0907444904019158. 15572765

[88] Liebschner D., Afonine P. V., Baker M.L., Bunkoczi G., Chen V.B., Croll T.I., Hintze B., Hung L.W., Jain S., McCoy A.J., (2019). Macromolecular structure determination using X-rays, neutrons and electrons: Recent developments in Phenix. Acta Crystallogr. D Struct. Biol. 75, 861–877. 10.1107/S2059798319011471. 31588918 PMC6778852

[89] Meng E.C., Goddard T.D., Pettersen E.F., Couch G.S., Pearson Z.J., Morris J.H., and Ferrin T.E. (2023). UCSF ChimeraX: Tools for structure building and analysis. Protein Science 32. 10.1002/pro.4792. PMC10588335 37774136

[90] Manalastas-Cantos K., Konarev P. V., Hajizadeh N.R., Kikhney A.G., Petoukhov M. V., Molodenskiy D.S., Panjkovich A., Mertens H.D.T., Gruzinov A., Borges C., (2021). ATSAS 3.0: Expanded functionality and new tools for small-angle scattering data analysis. J. Appl. Crystallogr. 54, 343–355. 10.1107/S1600576720013412. 33833657 PMC7941305

[91] Konarev P. V, Volkov V. V, Sokolova A. V, Koch M.H.J., and Svergun D.I. (2003). PRIMUS: a Windows PC-based system for small-angle scattering data analysis. ¯ PRIMUS J. Appl. Cryst 36, 1277–1282.

[92] Guinier A. (1939). Small-angle X-ray diffraction: application to the study of ultramicroscopic phenomena. Ann. Phys. (Paris). 11, 161–237.

[93] Svergun D.I. (1992). Determination of the regularization parameter in indirect-transform methods using perceptual criteria. J. Appl. Crystallogr. 25, 495–503. 10.1107/S0021889892001663.

[94] Svergun D., Barberato C., and Koch M.H.J. (1995). CRYSOL-a Program to Evaluate X-ray Solution Scattering of Biological Macromolecules from Atomic Coordinates.

[95] Bernadó P., Mylonas E., Petoukhov M. V., Blackledge M., and Svergun D.I. (2007). Structural characterization of flexible proteins using small-angle X-ray scattering. J. Am. Chem. Soc. 129, 5656–5664. 10.1021/ja069124n. 17411046

[96] Tria G., Mertens H.D.T., Kachala M., and Svergun D.I. (2015). Advanced ensemble modelling of flexible macromolecules using X-ray solution scattering. IUCrJ 2, 207–217. 10.1107/S205225251500202X. PMC4392415 25866658

[97] Ishizaki K., Nishihama R., Ueda M., Inoue K., Ishida S., Nishimura Y., Shikanai T., and Kohchi T. (2015). Development of gateway binary vector series with four different selection markers for the liverwort marchantia polymorpha. PLoS One 10. 10.1371/journal.pone.0138876. PMC4583185 26406247

[98] Ishizaki K., Chiyoda S., Yamato K.T., and Kohchi T. (2008). Agrobacterium-mediated transformation of the haploid liverwort Marchantia polymorpha L., an emerging model for plant biology. Plant Cell Physiol. 49, 1084–1091. 10.1093/pcp/pcn085. 18535011

[99] Kodama Y. (2016). Time gating of chloroplast autofluorescence allows clearer fluorescence imaging in planta. PLoS One 11. 10.1371/journal.pone.0152484. PMC4814121 27027881

[100] Kikhney A.G., Borges C.R., Molodenskiy D.S., Jeffries C.M., and Svergun D.I. (2020). SASBDB: Towards an automatically curated and validated repository for biological scattering data. Protein Science 29, 66–75. 10.1002/pro.3731. 31576635 PMC6933840

