# Supplementary material for: Structure determination and dual targeting of a plant TACO1 identifies its ancient role as an organelle translation regulator
Source: bioRxiv. 2026 Jun 1:2026.05.28.728419. Preprint. [Version 1] doi: 10.64898/2026.05.28.728419 (PMC13252137; doi:10.64898/2026.05.28.728419)
Supplement: Supplement 1 [file media-1.pdf]

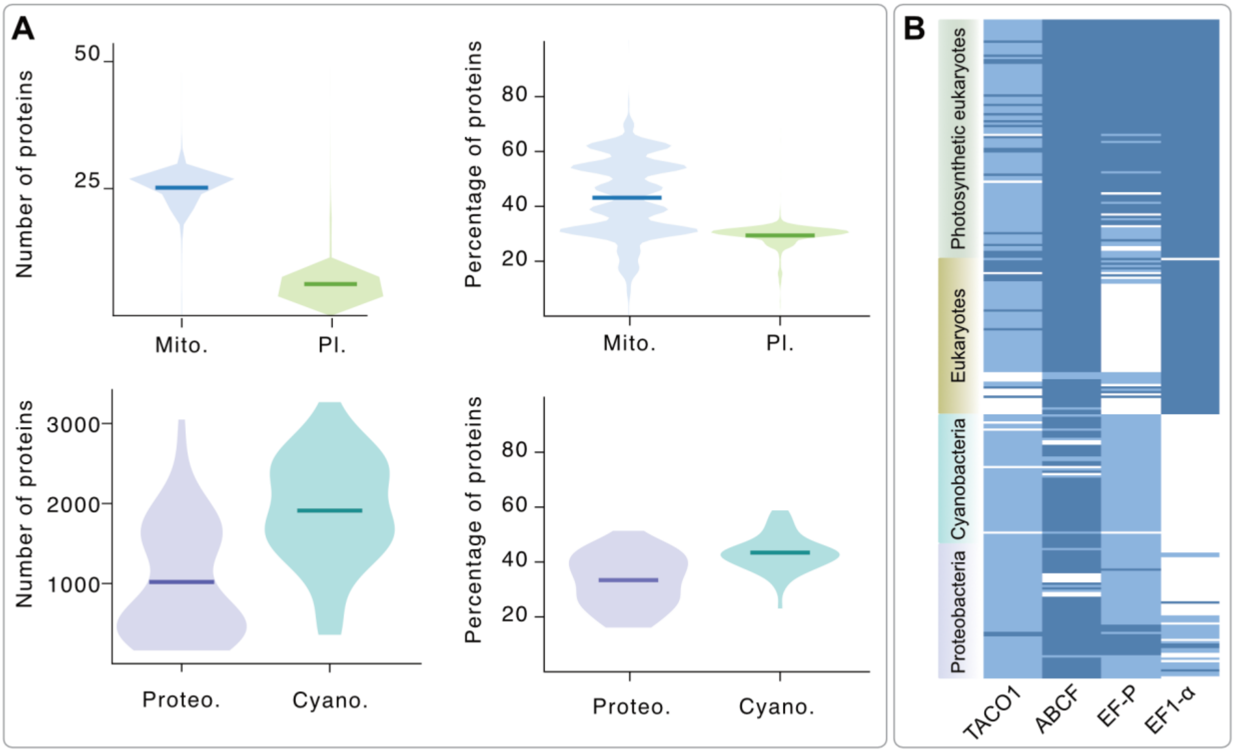

Fig. S1: Prevalence of polyproline motifs and ribosomal rescue factors across species. (A) Number and percentage of proteins with at least one polyproline (PP) motif across 18,059 mitochondrial, 15,208 plastid, 57 proteobacterial, and 56 cyanobacterial genomes. **(B)** Distribution of ribosomal rescue factors across 169 eukaryotic, 57 proteobacterial and 56 cyanobacterial genomes. White, no homologue; blue, single homolog; dark blue, multiple homologs.

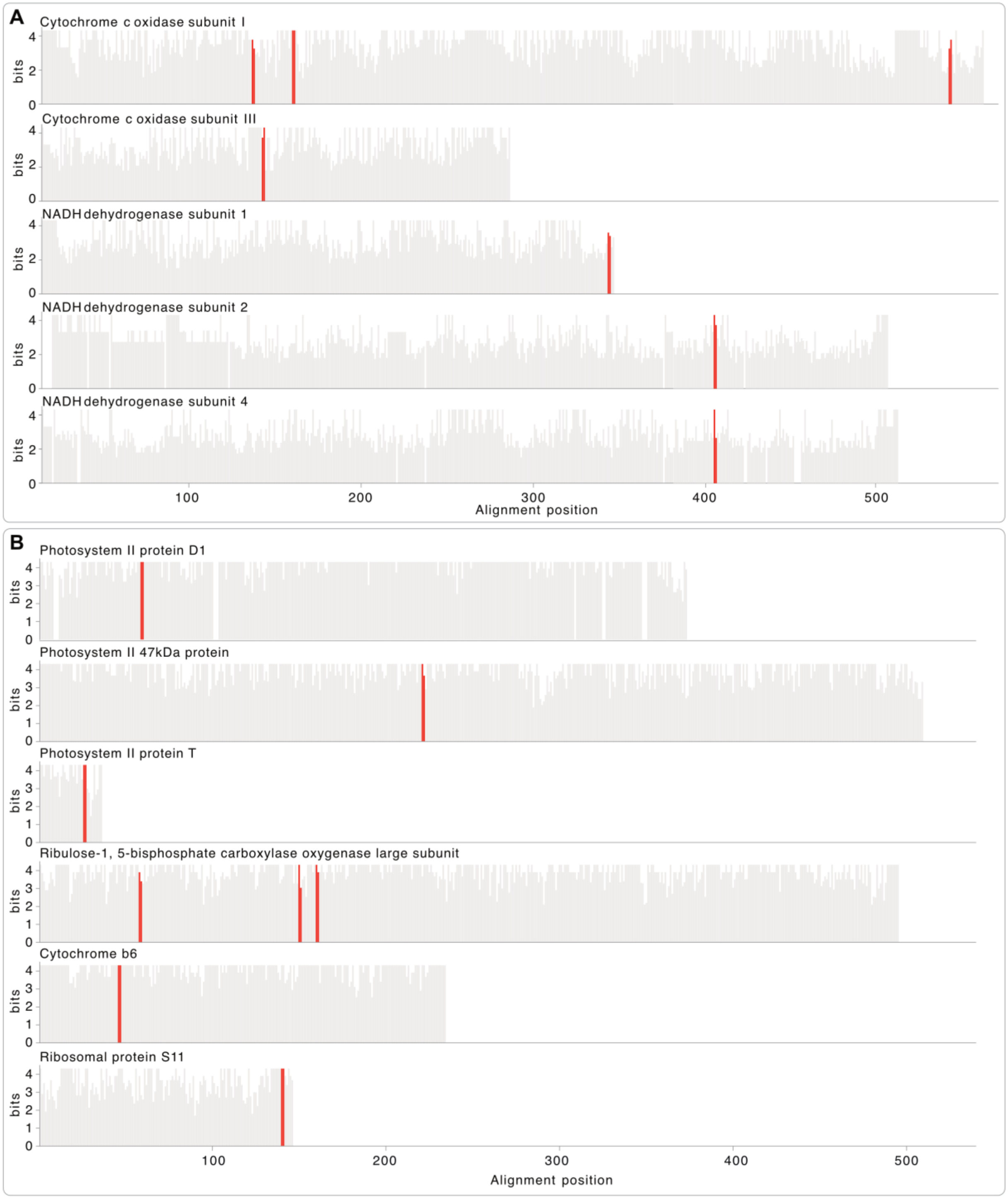

Fig. S2: Conservation of polyproline motifs across species. (A) Amino acid conservation across key proteins from seven mitochondrial and *Rickettsia prowazekii* (a phylogenetic relative of the mitochondrial ancestor), and **(B)** across ten plastid and *Synechococcus elongatus* (a representative cyanobacterial genome) genomes. Each bar shows the conservation of a single residue in the alignment as bit score (4 or higher = single amino acid is conserved across all species; 2, moderate conservation; 0, near-random amino acid is tolerated in that position). Red bars represent two subsequent prolines.

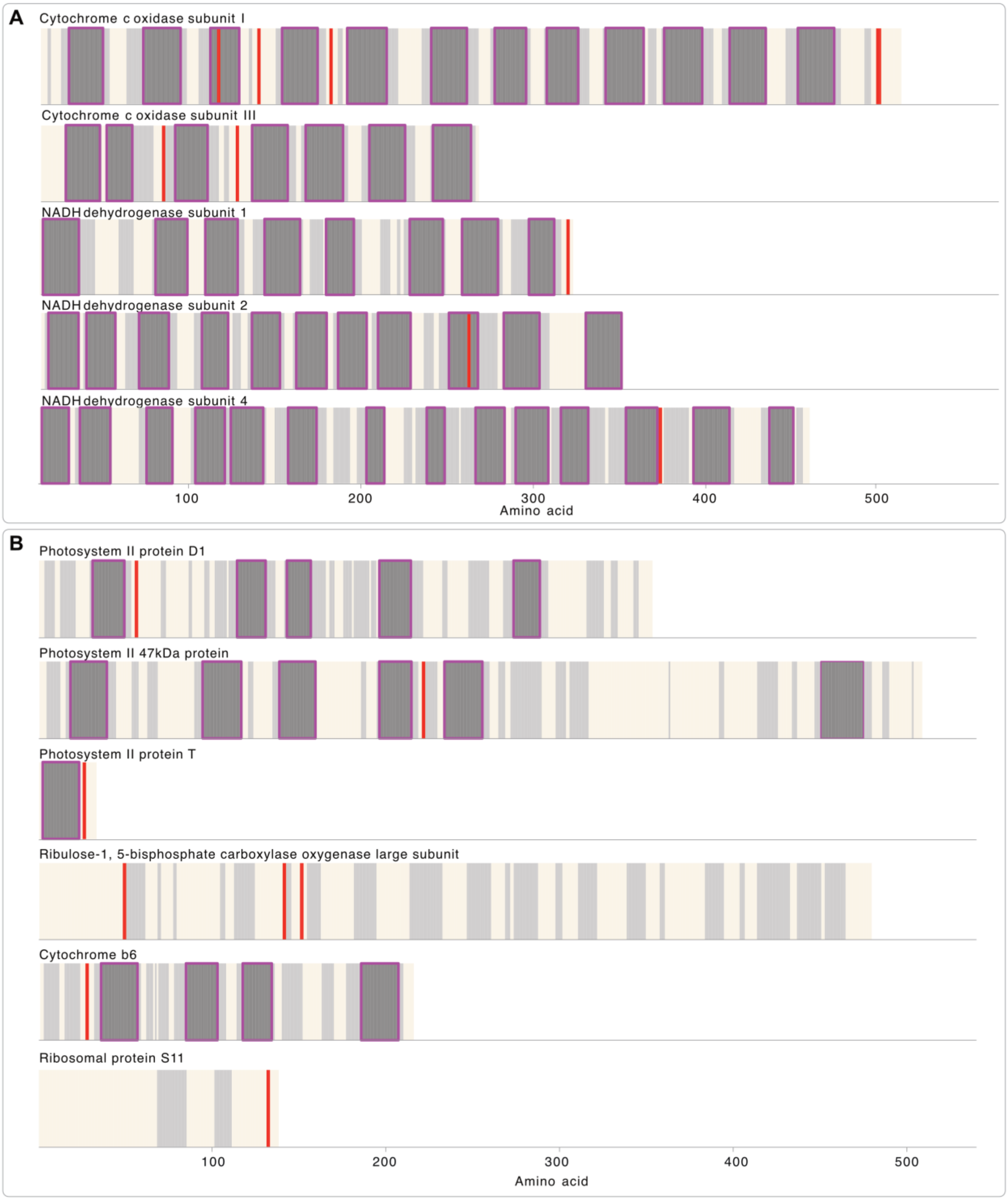

Fig. S3: Polyproline motifs in the context of protein secondary structure of organelle encoded proteins. (A) Secondary structure prediction for key genes from human mitochondrial and **(B)** Arabidopsis plastid genomes. Amino acids that are predicted to be inside an alpha helix (NetSurf3) are shown in light grey, those inside a membrane helix (deepTMHMM) are in dark grey (highlighted by violet boxes), poly-prolines in red, and other amino acids in off-white.

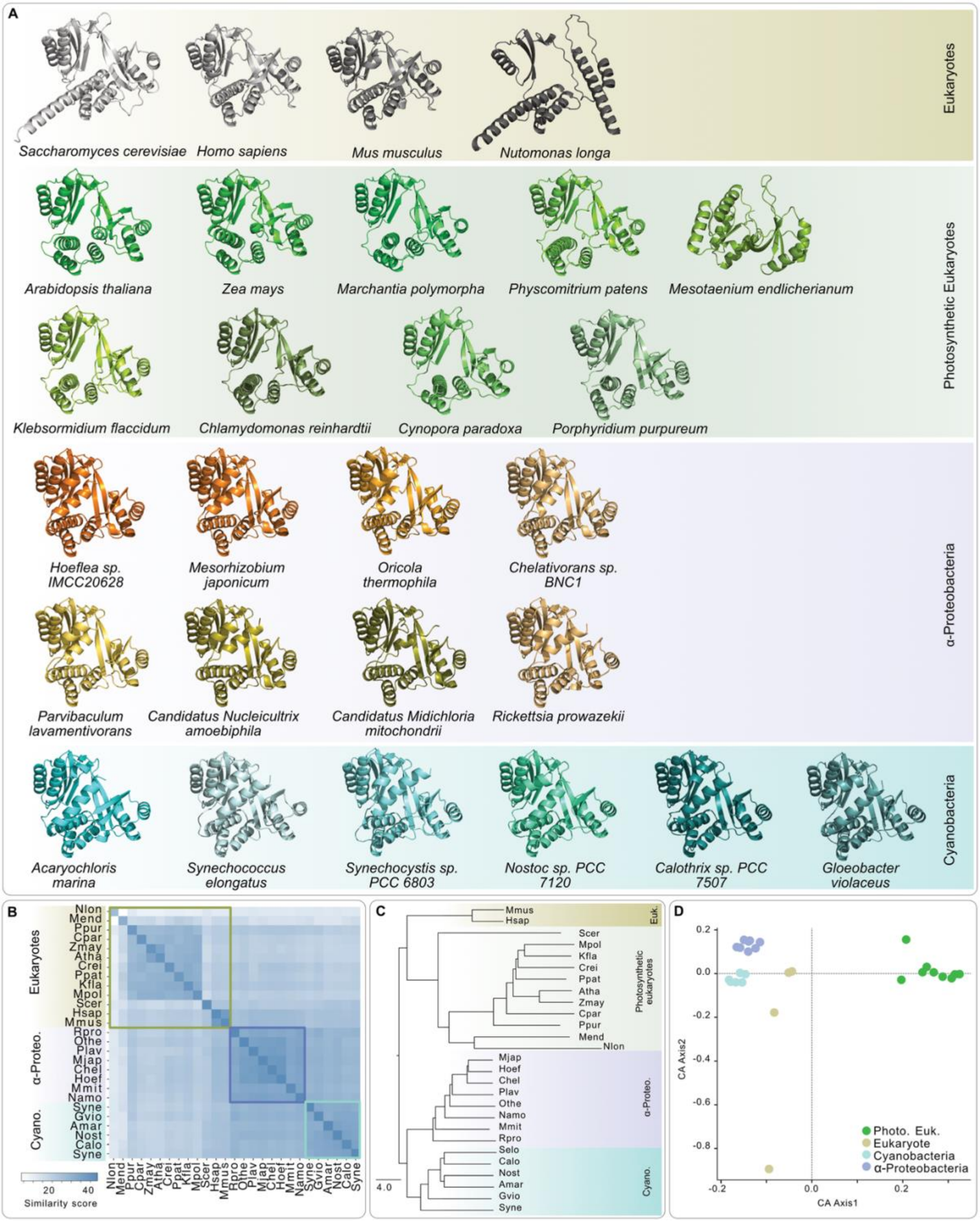

Fig. S4: Structural conservation of TACO1 homologs across species. (A) Predicted structures (AlphaFold 3) of TACO1/yebC homologues from eukaryotes and prokaryotes, **(B)** their DALI (Holm et al. 2020) similarity matrix, **(C)** structural similarity dendrogram rooted between prokaryotes and eukaryotes, **(D)** and a correspondence analysis that clusters similar structures together.

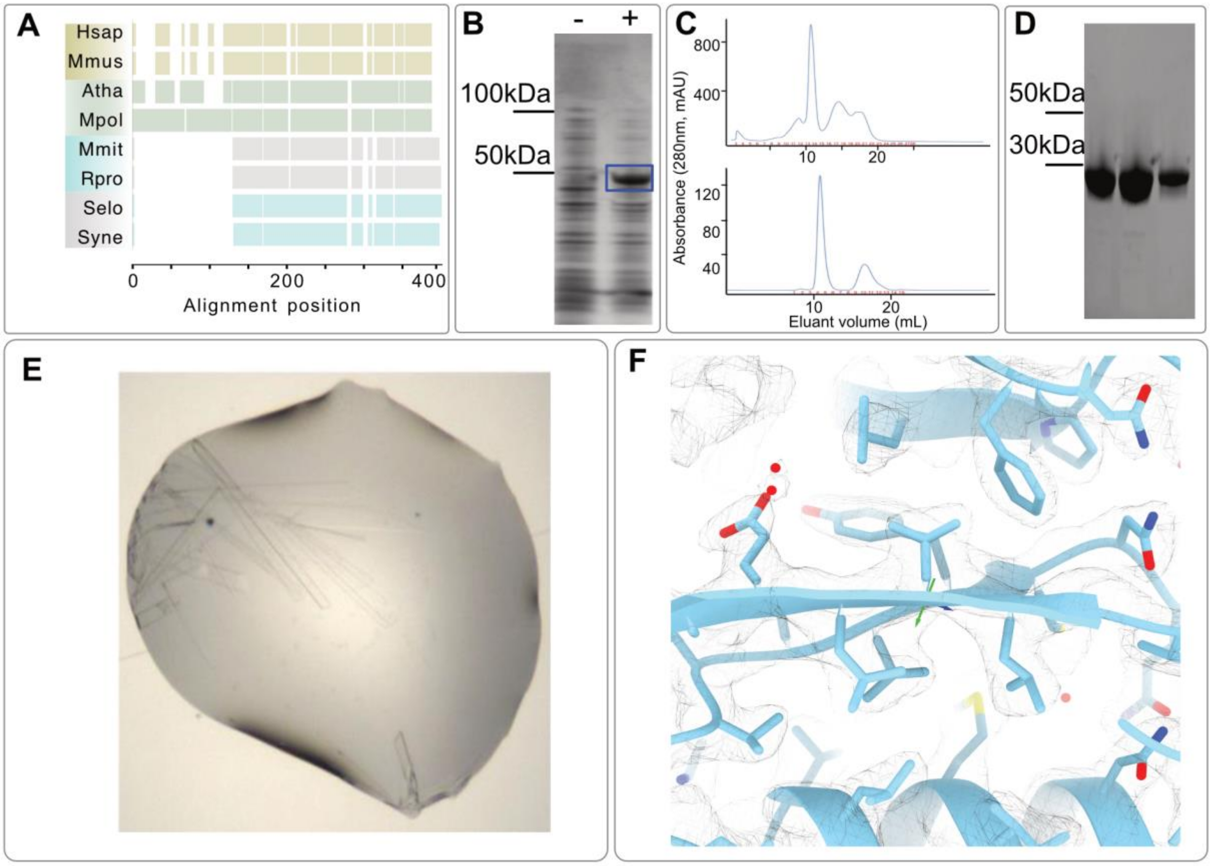

Fig. S5: Heterologous expression, purification and crystallization of MpTACO1. (A) Multiple sequence alignment of four eukaryotic TACO1 and four prokaryotic yebC homologs highlight the length of the targeting peptides (TP) that were added during evolution to the nuclear-encoded homologs. MpTACO1 (Mp1g23800; amino acids 127-374) N-terminally fused with 6X-Histidine and SUMO, and expressed in *E. coli* without (-) and with (+) inducer; total protein from overnight culture separated on SDS-PAGE and visualized with Coomassie Brilliant Blue (the blue box indicates expected protein band for MpTACO1). **(B-C)** Anion exchange chromatogram with major peaks in fraction 13-15 and size exclusion (Superdex™ 75 10/300 GL) chromatogram with major peaks in fractions 4-6, **(D)** which are resolved on SDS-PAGE and visualized with Coomassie Brilliant Blue. **(E)** Plates formed after 20h of crystallization in the Hcryo screen (crystallization solution: 0.1M MES, pH 6, 30% (v/v) polyethylene glycol 200, 5% (w/v) polyethylene glycol 3350). (**F)** Close up of the atomic modelling of MpTACO1 into the electron density. Density is shown as a mesh at 1 standard deviations (σ) above the mean.

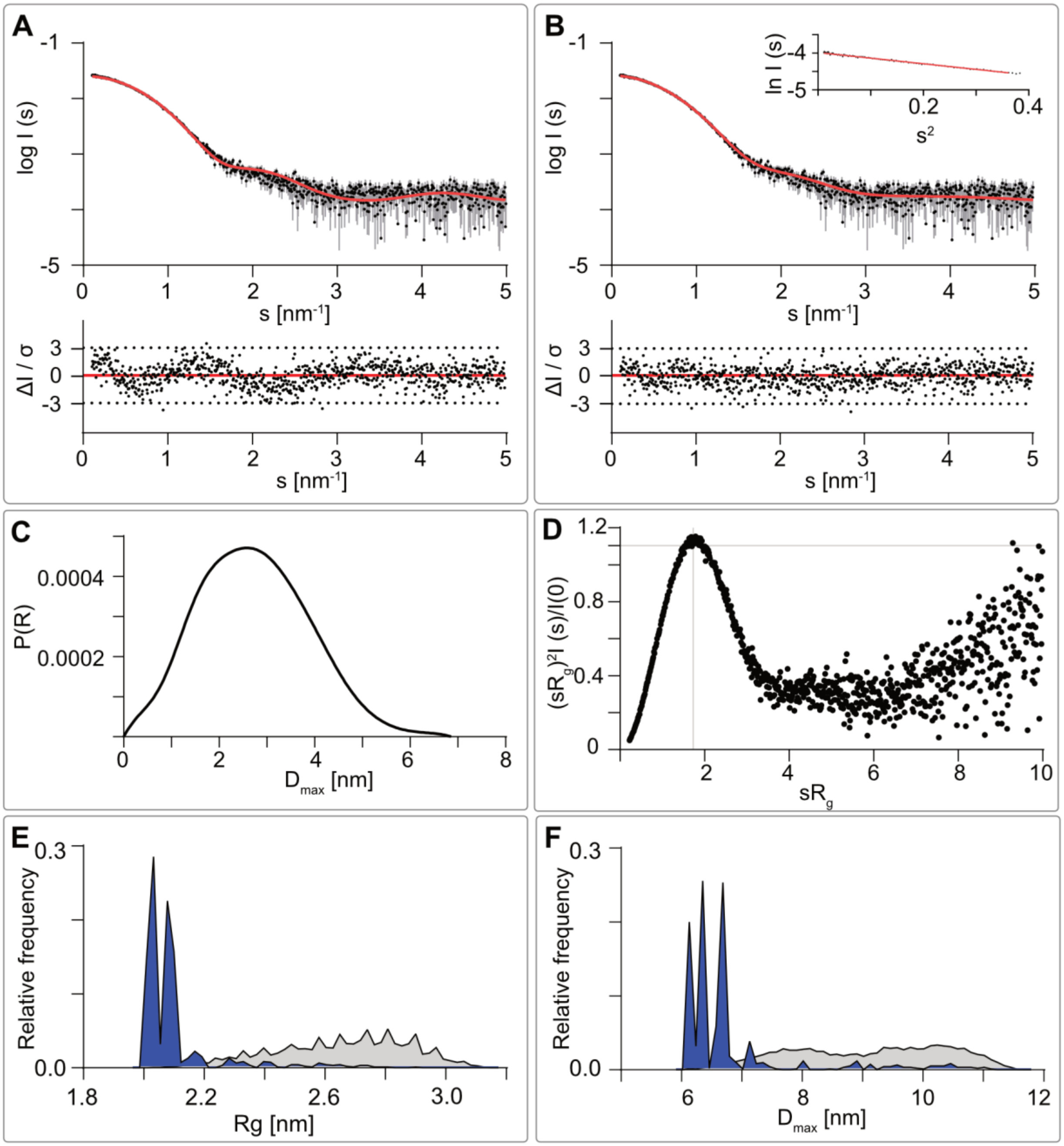

Fig. S6: Small-angle X-ray scattering data of MpTACO1. (A) Experimental data (black dots, with grey error bars) and the theoretical scattering intensity (red line) from the initial crystal structure calculated with CRYSOL (χ^2 value 1.39). Shown below is the residual plot of the data. **(B)** Experimental data (black dots, with grey error bars) and the EOM ensemble (red line) along with the residual plot of the data at bottom. The Guinier plot is added in the top right corner. **(C)** The Distance distribution function *(p(r)* function) indicating a globular molecule with small elongation. **(D)** Dimensionless Kratky plot indicating a compact molecule with certain degree of flexibility. **(E)** R_g and **(F)** D_{max} distributions calculated by EOM. Random pool distribution is shown in grey and the selected models in blue.

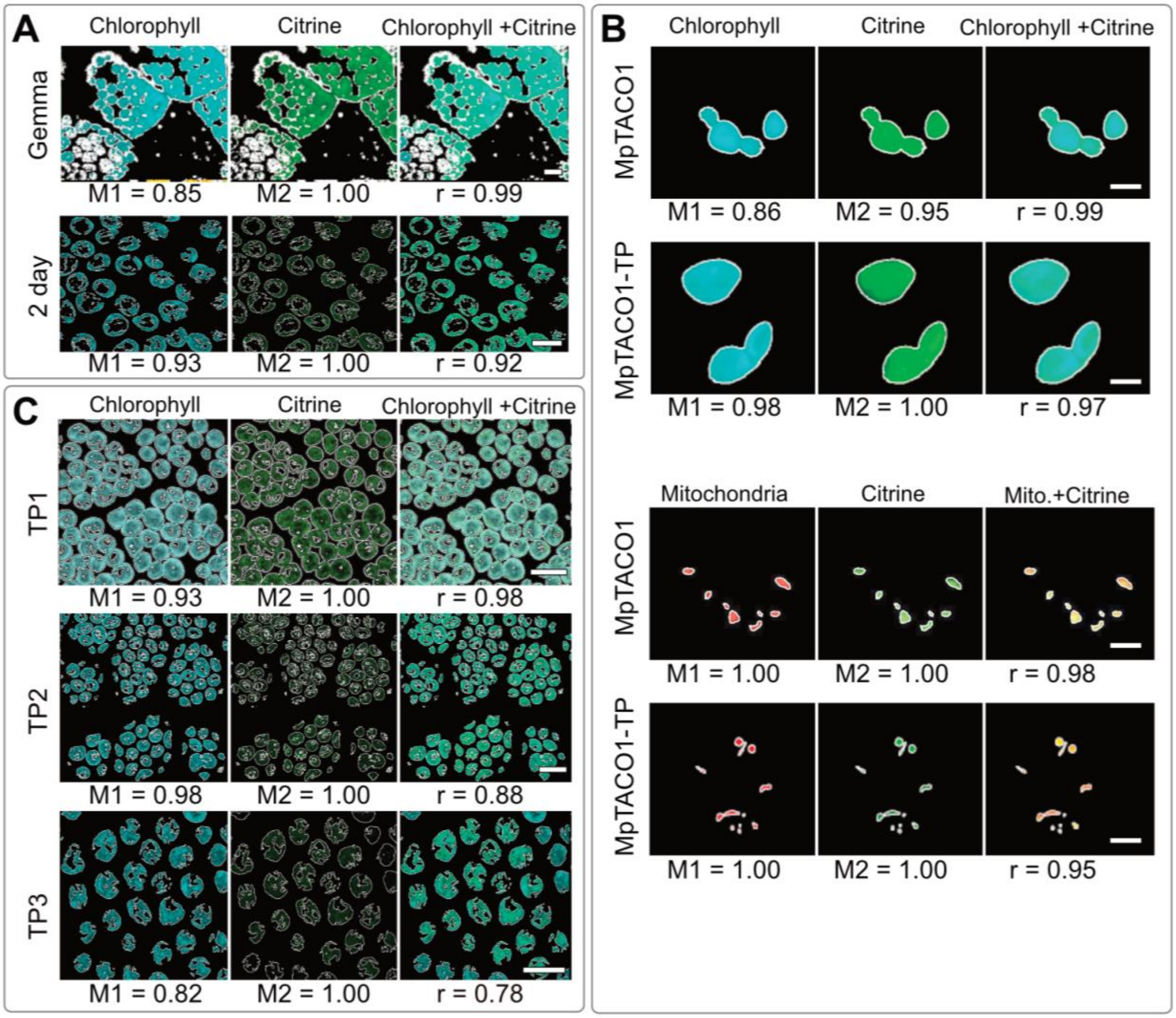

Fig. S7: Co-localization analysis on plant lines expressing MpTACO1:Citrine, MpTACO1-TP:Citrine and variants therein. Colocalization analyses on each image from the main figure 3. Mander's overlap coefficients M1 and M2, respectively, show the fraction of the organelle signal overlapping with the reporter and *vice versa*; e.g. in the first figure in S7A, M1=0.85 indicate that 85% of chlorophyll signal overlaps with GFP and M2=1 indicates that 100% of GFP overlaps with chlorophyll. R shows the correlation coefficient for intensities of the two channels. Scalebars 10µm.

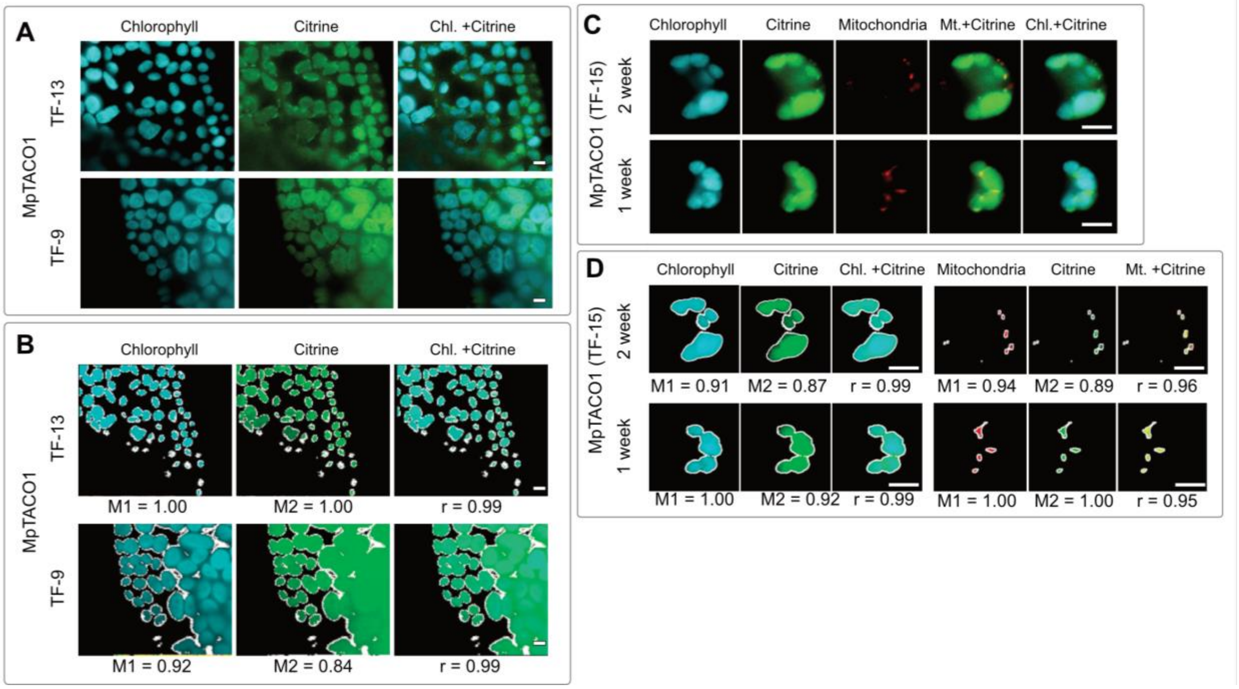

Fig. S8: Additional microscopy images and co-localization analyses supporting the dual localization of MpTACO1. (A) Intracellular localisation of MpTACO1 in thalli of two independent transfectant lines with the corresponding colocalization analyses in **(B)**. **(C)** Images of protoplasts generated from one- and two-week-old thalli of transfectant lines expressing full MpTACO1 with the corresponding colocalization analyses in **(D)**. Plastids were imaged through their autofluorescence and protoplast mitochondria through MitoTracker Red. Scalebars 10µm.

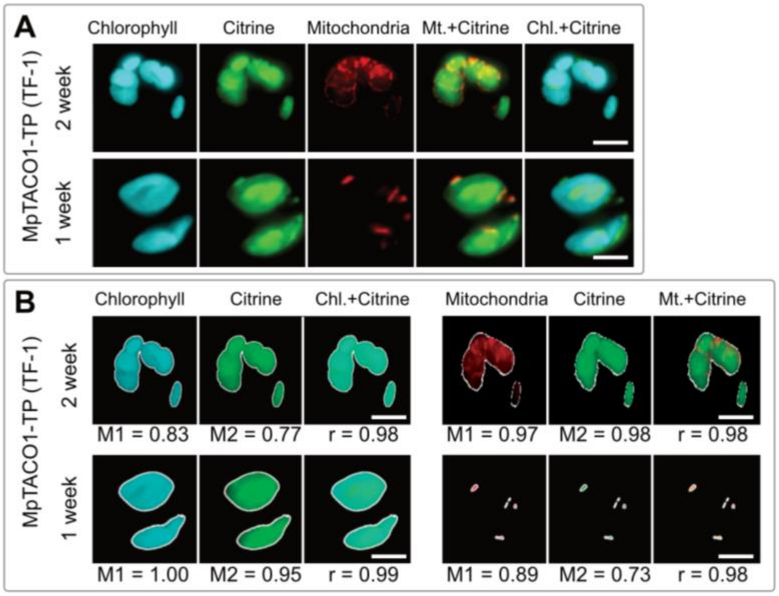

Fig. S9: Additional microscopy images and co-localization analyses for MpTACO-TP. Additional images of protoplast from thalli of MpTACO1-TP transfectants **(A)** and colocalization analyses **(B)**. Plastids were imaged through their autofluorescence and protoplast mitochondria through MitoTracker Red. Scalebars 10µm.

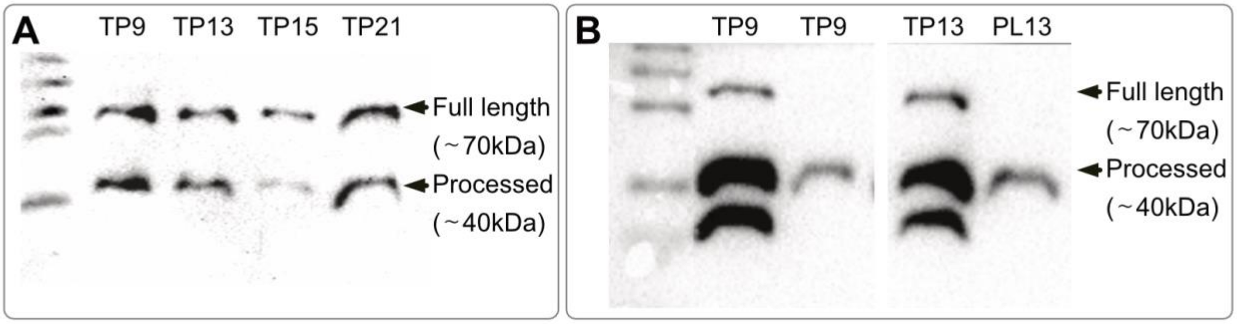

Fig. S10: Immunoblotting from plant lines expressing MpTACO1:citrine. Immunoblotting against citrine from total protein (TP) of four independent TACO1:citrine overexpression lines **(A)** and from total protein and isolated plastid fraction (PL) of two independent lines **(B)**.

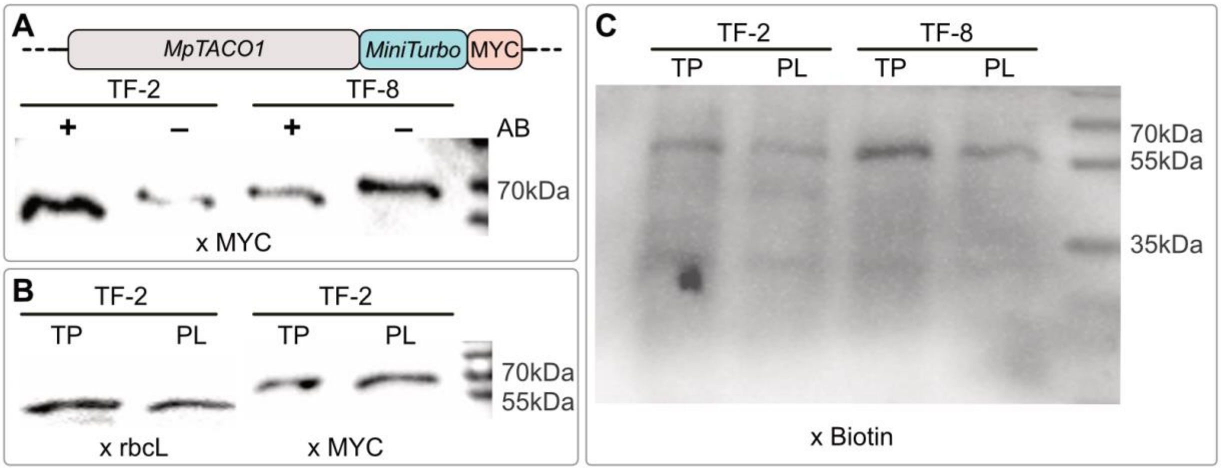

Fig. S11: Expression and localisation of MpTACO1 fused to miniTurbo. (A) Immunoblotting (against MYC tag) from total protein of 10-day-old thalli of two independent transfectant lines grown with (+) and without (-) antibiotic. **(B)** Immunoblotting (against rbcL and MYC tag) from total protein (TP) and plastid protein (PL) of 10-day-old thalli. **(C)** Immunoblotting (against biotin) from total protein (TP) and plastid protein (PL) of 10-day-old thalli from two independent transfectant lines, after 24h of biotin treatment.

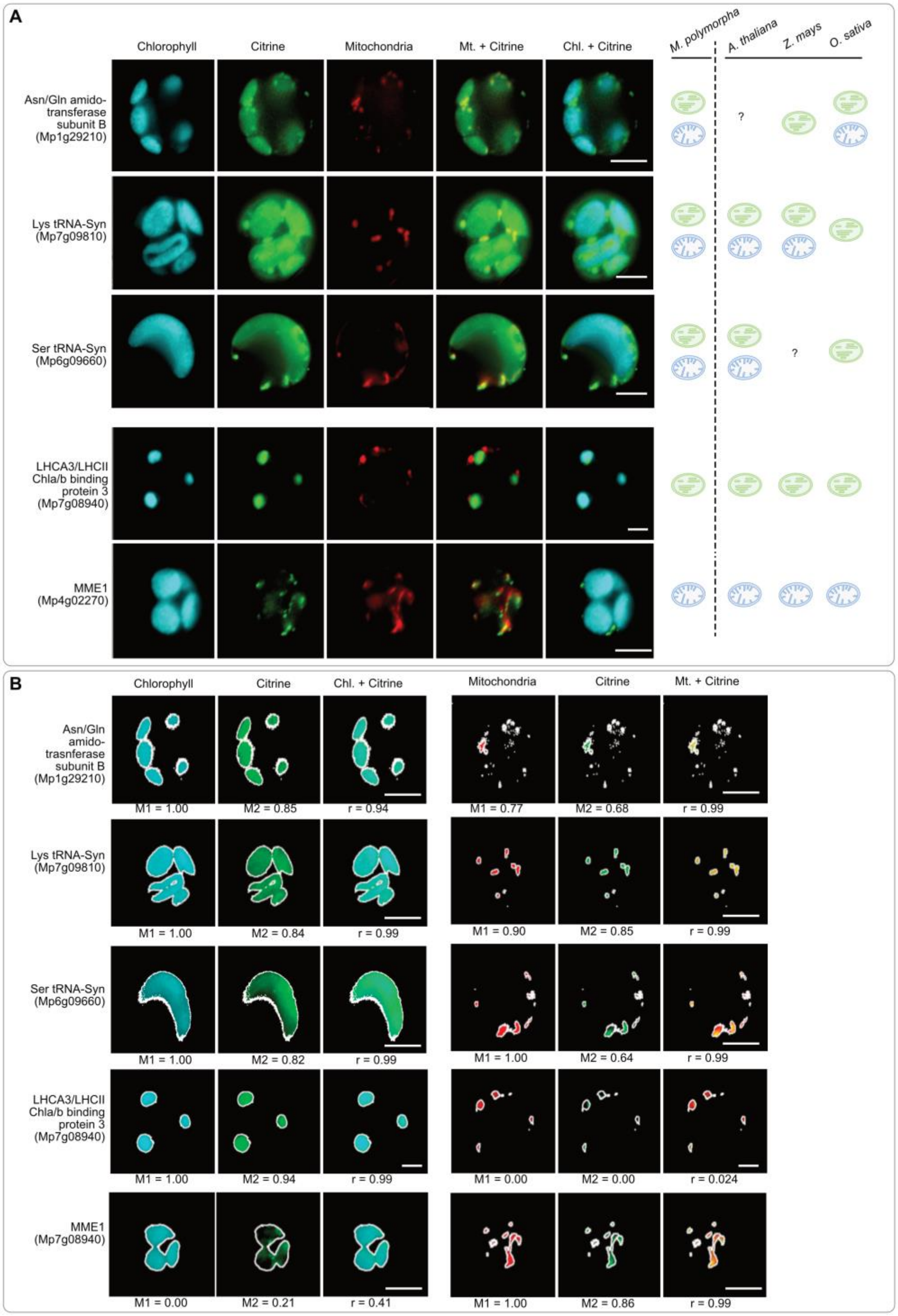

Fig. S12: Dual targeting of three information processing-related proteins. **(A)** Localization of three information processing-related candidates fused to citrine, along with plastid- and mitochondria-only controls, in protoplasts generated from thalli of one week old isogenic plant lines. Localization of homologs of these proteins, across four species, is depicted on the left. **(B)** Co-localization analyses on the same images. Plastids were imaged through their autofluorescence and protoplast mitochondria through MitoTracker Red. Scalebars 10μm.

Supplementary Table S1: X-ray data collection and refinement statistics for MpTACO1

Data collection statistics	
Beamline	ID30A-3
Space group	C 1 2 1
Cell dimensions a, b, c (Å)	91.7, 38.5, 78.1
Cell dimensions α, β, γ (°)	90, 115.6, 90
Resolution range (Å)	70.49-2.34
Wavelength (Å)	0.9677
R_{merge}	0.157 (1.191)[a]
R_{meas}	0.184 (1.388)
R_{pim}	0.095 (0.710)
I/σ(I)	7.4 (1.2)
$CC_{1/2}$	0.993 (0.721)
Completeness (%)	97.61 (98.03)
Multiplicity	7.1 (7.2)
Wilson B-factor (Å^2)	42.65
Total number of observations	73,568 (7,298)
Total number unique	10,404 (2,582)
Refinement statistics	
Resolution (Å)	45.18-2.34 (2.57-2.34)
R_{work}/R_{free} (%)	23.31/27.90
Reflections used for R_{free}	510
Number of atoms protein/water	3,722/28
Average B-factor	50.04
R.m.s. deviations[b]	
Bond lengths (Å)	0.003
Bond angles (°)	0.62
Ramachandran analysis	
Favoured region (%)	99.15
Allowed region (%)	0.85
Outliers (%)	0.0
Clashscore	1.08
Rotamer outliers (%)	0.0

Supplementary Table S2: SAXS Data.

**Data collection parameters**	
SAXS Device	Xenocs Xeuss 2.0 with Q-Xoom
Detector	PILATUS 3 R 300K windowless
Detector distance (m)	0.55
Beam size	0.8 mm x 0.8 mm
Wavelength (nm)	0.154
Sample environment	Low Noise Flow Cell, 1 mm ø
Absolute scaling method	Comparison with scattering from pure H_2O
Normalization	To transmitted intensity by direct beam-
Scattering intensity scale	Absolute scale, cm^{-1}
s range (nm^{-1})	0.05 – 5.5
**Sample**	**MpTACO1**
Organism	*Marchantia polymorpha*
UniProt ID	9SCZ
Mode of measurement	batch
Temperature (°C)	15
Exposure time (# frames)	600 s (36 frames)
Protein buffer	50 mM Tris-HCl pH 8.0, 150 mM NaCl
Protein concentration (mg/ml)	5.44
**Structural parameters**	
Guinier Analysis (PRIMUS)	
$I(0) \pm \sigma$ (cm^{-1})	0.019 ± 0.00005
$R_g \pm \sigma$ (nm)	2.14 ± 0.010
s-range (nm^{-1})	0.105 – 0.602
min < sRg < max limit	0.225 – 1.288
Data point range	1 - 85
Linear fit assessment (R^2)	0.992
PDDF/P(r) Analysis (GNOM)	
$I(0) \pm \sigma$ (cm^{-1})	0.018 ± 0.00005
$R_g \pm \sigma$ (nm)	2.10 ± 0.007
D_{max} (nm)	6.86
Porod volume (nm^3)	45.20
s-range (nm^{-1})	0.105 – 5.356
χ2 / CorMap P-value	1.049 / 0.585
**Molecular mass (kDa)**	
From *I*(0)	26.31
From Qp [1]	24.07
From MoW2 [2]	27.04
From Vc [3]	28.62
From Bayesian Inference [4]	26.23
From GNNOM [5]	27.30
From sequence	26.98 (monomer)
**Atomistic modelling**	
EOM	

Symmetry	P1
s-range for fit (nm^{-1})	0.105 – 4.988
χ^2, CorMap P-value	1.024 / 0.808
**SASBDB accession codes** [6]	SASDYA7
**Software**	
ATSAS Software Version [7]	3.0.5 (ATSAS online 3.2.1)
Primary data reduction	PRIMUS [8]
Data processing	GNOM [9]
Flexibility ensemble modelling	EOM [10,11]
Statistic goodness-of-fit test	χ^2, CorMap [12]

References

1. Porod, G. Die Röntgenkleinwinkelstreuung Von Dichtgepackten Kolloiden Systemen - 1 Teil. *Kolloid-Zeitschrift and Zeitschrift Fur Polymere* 124, 83–114 (1951).

2. Fischer, H., De Oliveira Neto, M., Napolitano, H. B., Polikarpov, I. & Craievich, A. F. Determination of the molecular weight of proteins in solution from a single small-angle X-ray scattering measurement on a relative scale. *J. Appl. Crystallogr.* 43, 101–109 (2010).

3. Rambo, R. P. & Tainer, J. A. Accurate assessment of mass, models and resolution by small-angle scattering. *Nature* 496, 477–481 (2013).

4. Hajizadeh, N. R., Franke, D., Jeffries, C. M. & Svergun, D. I. Consensus Bayesian assessment of protein molecular mass from solution X-ray scattering data. *Sci. Rep.* 8, (2018).

5. Molodenskiy, D. S., Svergun, D. I. & Kikhney, A. G. Artificial neural networks for solution scattering data analysis. *Structure* 30, 900-908.e2 (2022).

6. Kikhney, A. G., Borges, C. R., Molodenskiy, D. S., Jeffries, C. M. & Svergun, D. I. SASBDB: Towards an automatically curated and validated repository for biological scattering data. *Protein Science* 29, 66–75 (2020).

7. Manalastas-Cantos, K. *et al.* ATSAS 3.0: Expanded functionality and new tools for small-angle scattering data analysis. *J. Appl. Crystallogr.* 54, 343–355 (2021).

8. Konarev, P. V, Volkov, V. V, Sokolova, A. V, Koch, M. H. J. & Svergun, D. I. PRIMUS: a Windows PC-based system for small-angle scattering data analysis. ¯*PRIMUS J. Appl. Cryst* 36, 1277–1282 (2003).

9. Svergun, D. I. Determination of the regularization parameter in indirect-transform methods using perceptual criteria. *J. Appl. Cryst* 25, 495–503 (1992).

10. Bernadó, P., Mylonas, E., Petoukhov, M. V., Blackledge, M. & Svergun, D. I. Structural characterization of flexible proteins using small-angle X-ray scattering. *J. Am. Chem. Soc.* 129, 5656–5664 (2007).

11. Tria, G., Mertens, H. D. T., Kachala, M. & Svergun, D. I. Advanced ensemble modelling of flexible macromolecules using X-ray solution scattering. *IUCrJ* 2, 207–217 (2015).

12. Franke, D., Jeffries, C. M. & Svergun, D. I. Correlation Map, a goodness-of-fit test for one-dimensional X-ray scattering spectra. *Nat. Methods* 12, 419–422 (2015).
